# T cells in type 1 diabetes: mechanisms of β-cell autoimmunity, innate-adaptive immune crosstalk, and translational immunotherapy

**DOI:** 10.3389/fimmu.2026.1845336

**Published:** 2026-10-19

**Authors:** Bushra Amer, Mohammed Alnukhali, Albaraa Haidarah, Anis Ahmad

**Affiliations:** 1 Department of Internal Medicine, Berkshire Medical Center, Pittsfield, MA, United States; 2 Department of Radiation Oncology, University of Miami Miller School of Medicine, Sylvester Comprehensive Cancer Center, Miami, FL, United States; 3 Department of Biochemistry and Molecular Biology, University of Miami Miller School of Medicine, Sylvester Comprehensive Cancer Center, Miami, FL, United States; 4 Faculty of Medicine, Al-Azhar University, Cairo, Egypt

**Keywords:** adaptive immunity, immune crosstalk, immunotherapy, innate immunity, precision medicine, regulatory T cells, T cells, type 1 diabetes

## Abstract

Type 1 diabetes (T1D) is an autoimmune disease in which immune responses drive the destruction of insulin-producing pancreatic β-cells, ultimately resulting in lifelong insulin dependence. Although T cells are central mediators of β-cell autoimmunity, growing evidence shows that innate immune cells, including dendritic cells, macrophages, and natural killer (NK) cells, play essential roles in initiating, shaping, and sustaining pathogenic adaptive responses within the islet microenvironment. Both CD4^+^ helper and CD8^+^ cytotoxic T lymphocytes contribute to β-cell injury through the production of inflammatory cytokines, direct cytotoxicity, and disruption of self-tolerance. At the same time, regulatory T cells exhibit reduced numbers and/or impaired suppressive function, thereby further accelerating autoimmune progression. Recent advances in animal models, single-cell transcriptomics, spatial immune profiling, and T cell receptor sequencing have refined our understanding of autoreactive repertoires, antigen specificity, and bidirectional crosstalk between innate and adaptive immune compartments. At the translational level, immune signatures are emerging as tools for disease prediction and patient stratification, while therapeutic strategies ranging from anti-CD3 monoclonal antibodies to tolerogenic vaccines and adoptive Treg therapies are moving from preclinical development into clinical evaluation. The clinical success of teplizumab as the first disease-modifying therapy for T1D reinforces the value of targeting immune pathways, but durable remission across diverse patient groups remains a major challenge. This review synthesizes recent advances in the immunobiology of T cells in T1D, emphasizing their functional interplay with innate immune cells, tracing the path from mechanistic discovery to clinical translation, and highlighting future directions for precision immunotherapy.

## 1 Introduction

T1D is an autoimmune disease of the pancreas characterized by immune-mediated destruction of insulin-producing β-cells (1, 2). T1D affects more than 8 million people worldwide and occurs across the lifespan, with a substantial proportion of incident and prevalent cases occurring in adults (3, 4). Despite advances in insulin therapy and glycosylated hemoglobin (HbA1c) monitoring technologies, individuals with T1D remain at elevated risk for vascular complications, impaired quality of life, and premature mortality (5, 6). These persistent challenges underscore the need for disease-modifying treatments that target immune pathology rather than simply replacing insulin (7, 8).

Among the immune factors that contribute to T1D, T lymphocytes are central participants in disease onset and progression (9–11). Infiltration of pancreatic islets by CD4^+^ helper and CD8^+^ cytotoxic T cells is a hallmark of T1D and reflects recognition of β-cell autoantigens presented by MHC molecules (12, 13). Quantitative or functional defects in regulatory T cells (Tregs), which normally maintain immune tolerance, further exacerbate the loss of immune homeostasis in T1D (14, 15). Together, these findings place T cells at the center of T1D immunopathogenesis, disease-activity biomarkers, and therapeutic targeting (16, 17).

Recognition of T cells as pathogenic mediators has stimulated decades of translational research aimed at bridging laboratory discoveries and clinical application. Classical studies have defined autoreactive T cell repertoires, elucidated cytokine networks, and confirmed genetic predispositions—particularly HLA class II alleles—as major risk factors for T1D (18–20). More recent technological advances, including single-cell transcriptomics, T cell receptor sequencing, and spatial multi-omic profiling of human pancreatic tissue, have substantially improved the ability to define autoreactive T-cell states, tissue-specific immune interactions, and disease-associated cellular niches (11, 21). These observations have informed immunotherapies designed either to suppress pathogenic T-cell responses or to induce antigen-specific tolerance (22, 23).

Translation of T cell research into T1D therapy has achieved important but incomplete success. Early generalized immunosuppressive regimens demonstrated that immune regulation can preserve β-cell function, but they failed to achieve sustained remission and were associated with unacceptable toxicity (2, 24). More recent approaches have focused on targeted interventions, including anti-CD3 monoclonal antibodies, checkpoint modulators, and adoptive Treg therapies (7, 22). A major milestone occurred in 2022, when the U.S. Food and Drug Administration approved teplizumab, an anti-CD3 antibody, demonstrating that modulation of pathogenic T-cell responses can delay progression to clinical T1D in at-risk individuals (25, 26). Nevertheless, durability of benefit, heterogeneity of patient response, and broad clinical implementation remain major concerns (25, 27).

As the field advances, the therapeutic goal is shifting from broad suppression of autoimmunity toward precise, durable, and safe restoration of immune tolerance (8, 9). Achieving this goal will require integration of immunogenomics, systems biology, and computational modeling to profile patients and tailor therapies to individual immunological states (22, 28). In addition, lessons from other autoimmune diseases and cancer immunotherapy provide useful parallels for the continued development of T cell–targeted strategies in T1D (29, 30).

Although autoreactive T cells are the principal effectors of β-cell destruction, accumulating evidence demonstrates that they do not act in isolation. Innate immune cells, including dendritic cells, macrophages, and NK cells, participate in the initiation, amplification, and persistence of pathogenic adaptive immune responses in T1D through antigen presentation, cytokine production, and inflammatory remodeling of the islet microenvironment (31, 32). This bidirectional innate–adaptive immune communication is increasingly recognized as a central feature of both disease initiation and progression, with important implications for immunotherapies aimed at restoring tolerance rather than merely suppressing autoreactive T cell populations (33, 34). Accordingly, this review integrates the mechanistic roles of T cells with their functional dependence on innate immune signals, tracing a path from immunopathogenesis to clinical translation with particular attention to therapeutic exploitation of this crosstalk.

This review summarizes current evidence on the role of T cells in the immunopathogenesis of T1D and its translation into clinical practice. We begin by outlining key mechanisms of T cell–mediated β-cell destruction and immune dysregulation, while also highlighting the interplay between adaptive and innate immune responses in the islet microenvironment. We then examine translational advances in biomarker discovery, HLA-associated risk, and experimental models that have improved understanding of disease heterogeneity and progression. In addition, we discuss established and emerging therapeutic strategies that modulate T cell responses, emphasizing their clinical promise and current limitations. Finally, we consider future directions in precision immunotherapy and the major challenges that must be addressed to translate mechanistic insights into durable, personalized interventions for T1D. Through this synthesis, we aim to provide a useful framework for clinicians, immunologists, and translational researchers working to advance immune-based strategies for T1D. An overview of T cell–driven β-cell autoimmunity and its translation into precision immunotherapy is presented in **Figure 1**.

**Figure 1:**
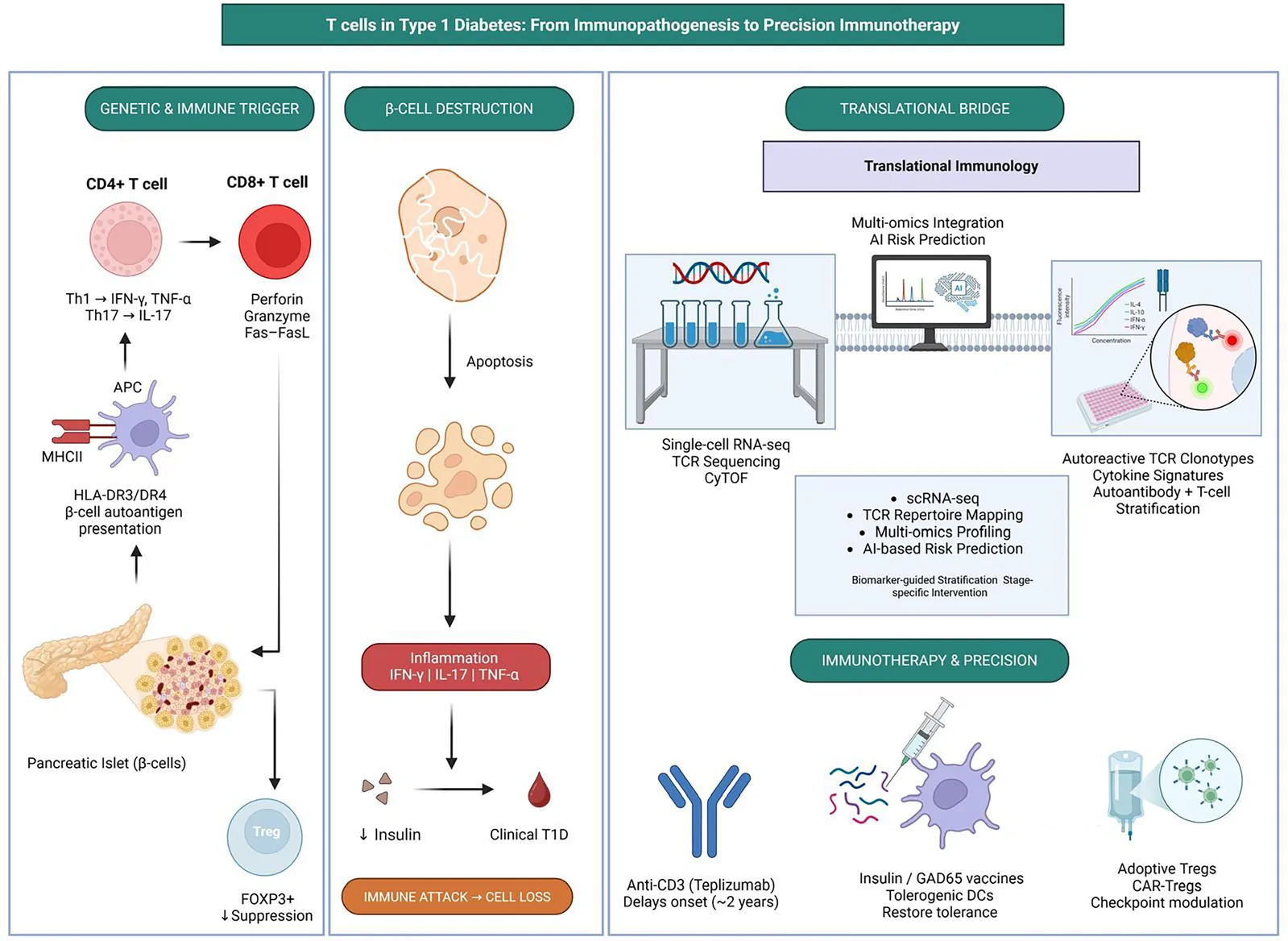
Overview of T cell–driven β-cell autoimmunity and its translation into precision immunotherapy in type 1 diabetes. This graphical summary illustrates how genetic susceptibility and immune dysregulation contribute to progressive β-cell loss in type 1 diabetes (T1D). HLA-DR3/DR4–associated antigen presentation promotes activation of autoreactive CD4^+^ helper and CD8^+^ cytotoxic T cells. CD4^+^ T cells amplify islet inflammation through Th1- and Th17-associated cytokines, including IFN-γ, TNF-α, and IL-17, whereas CD8^+^ T cells directly injure β-cells through perforin, granzyme, and Fas–FasL-mediated cytotoxic pathways. Reduced FOXP3+ regulatory T-cell suppressive function further weakens peripheral tolerance, allowing autoreactive responses to persist. Together, these processes drive β-cell apoptosis, inflammatory cytokine production, declining insulin secretion, and eventual clinical T1D. The translational panel highlights emerging tools for patient stratification and therapeutic development, including single-cell RNA sequencing, TCR sequencing, CyTOF, multi-omics integration, cytokine profiling, autoantibody assessment, and AI-assisted risk prediction. Current and investigational immune-based strategies include anti-CD3 therapy such as teplizumab, insulin- or GAD65-based antigen-specific vaccines, tolerogenic dendritic cells, adoptive Treg therapy, CAR-Tregs, and immune checkpoint modulation, all aimed at restoring immune tolerance and supporting more precise intervention. Created with BioRender.com.

## 2 T cell immunopathogenesis in type 1 diabetes

In this review, T-cell activation refers to antigen/TCR-driven acquisition of effector function, whereas T-cell-mediated β-cell destruction refers specifically to β-cell injury resulting from autoreactive T-cell effector mechanisms.

### 2.1 Environmental and contextual triggers of innate–adaptive immune activation in T1D

Although autoreactive T cells are central effectors of β-cell destruction, the initiation of T1D is strongly influenced by environmental and contextual factors that condition innate immune activation before overt adaptive autoimmunity is established (35, 36). Enteroviral infections, particularly coxsackievirus-associated β-cell injury, have been implicated in triggering local inflammation, type I interferon responses, and enhanced antigen presentation within pancreatic tissue (37, 38). Viral infection may increase β-cell stress and apoptosis, thereby expanding the pool of β-cell antigens available for uptake by dendritic cells and macrophages; these activated innate immune cells can then migrate to pancreatic lymph nodes, where they prime autoreactive CD4^+^ and CD8^+^ T cell responses in genetically susceptible individuals (39).

Gut microbiota dysbiosis also contributes to immune conditioning in T1D by altering intestinal barrier function, microbial metabolite production, and basal innate immune tone (40). Reduced microbial diversity, altered short-chain fatty acid production, and increased intestinal permeability may promote systemic inflammatory signaling and influence dendritic cell maturation, regulatory T cell stability, and Th1/Th17 polarization (40). Early-life exposures, including delivery mode, antibiotic use, breastfeeding patterns, and childhood infections, further shape immune education during a critical developmental window (41).

In parallel, dietary, metabolic, and environmental stressors can increase β-cell endoplasmic reticulum stress, oxidative injury, and inflammatory chemokine production, thereby enhancing β-cell visibility to the immune system and favoring neoantigen formation (37). Together, these environmental and metabolic inputs provide a mechanistic bridge between genetic susceptibility, innate immune priming, and subsequent autoreactive T cell activation (42). This broader framework supports the concept that T1D emerges not solely from autonomous T cell dysfunction but from the convergence of β-cell stress, innate immune sensing, and adaptive immune misdirection (36).

### 2.2 Pathogenic roles of CD4^+^ helper T cells

CD4^+^ T cells play a key role in orchestrating immune responses against pancreatic β-cells. These cells recognize β-cell antigens presented as HLA class II complexes on antigen-presenting cells (APCs), including dendritic cells, macrophages, and B cells (18, 19, 26). Following activation, CD4^+^ T cells differentiate into functional subsets that contribute to disease pathogenesis in distinct ways (9, 16, 26). Th1 cells release pro-inflammatory cytokines, including interferon-γ (IFN-γ) and tumor necrosis factor-α (TNF-α), that promote local inflammation and support cytotoxic killing by CD8^+^ T cells (10, 43). Th17 cells produce interleukin-17 (IL-17), which recruits innate immune cells and amplifies tissue inflammation, thereby contributing to β-cell stress and apoptosis. Human studies have identified increased Th17/IL-17-associated immune activity in peripheral blood and pancreatic islets from individuals with T1D (44). More recently, longitudinal single-cell RNA sequencing of peripheral CD4+ T cells from children who subsequently developed T1D identified early, cell-type-specific transcriptional changes across CD4+ T-cell subsets, including altered regulatory activity within Th17 cells, providing contemporary human evidence of CD4+ T-cell dysregulation before clinical disease (45).

Th2 cells are generally associated with type 2 immune responses and may counterbalance Th1-dominated inflammation in some contexts; however, their role in T1D is context-dependent, highlighting a more complex imbalance among T helper-cell subsets (8). In contrast, direct evidence for causality derives largely from experimental models. In NOD mice, depletion, functional perturbation, and adoptive-transfer experiments support a pathogenic role for CD4+ T cells and Th1-associated responses in β-cell destruction (46).

### 2.3 CD8^+^ cytotoxic T cells as direct mediators of β-cell destruction

CD8^+^ T cells are direct effectors of β-cell killing, whereas CD4^+^ T cells primarily orchestrate broader immunologic disequilibrium. These effector T cells recognize HLA class I-presented β-cell autoantigens and induce apoptosis through perforin/granzyme release and Fas–Fas ligand interactions (12, 47, 48).

Islet-infiltrating CD8^+^ T cells are among the earliest immune signals observed in both NOD mice and human autopsy samples (13, 48, 49). T cell receptor (TCR) studies have identified autoreactive CD8^+^ lymphocyte populations targeting insulin, glutamic acid decarboxylase (GAD65), islet antigen-2 (IA-2), and zinc transporter 8 (ZnT8) (48). Recent TCR-profiling studies have further characterized autoreactive CD8 T-cell clonotypes and their antigen specificity across T1D, supporting their potential value as biomarkers of disease-associated immune activity (11, 48). Recent single-cell analysis of peripheral blood from individuals with T1D further demonstrated autoantigen-responsive cytotoxic CD8+ T-cell populations with increased inflammatory gene expression following insulin stimulation, providing additional human evidence of disease-associated CD8+ T-cell activation (50).

Because CD8^+^ T cells are pathogenic effectors, therapeutic strategies targeting them are compelling. However, broad depletion may compromise antiviral immunity and other protective T cell functions. The key translational challenge is therefore to modulate autoreactive CD8^+^ T cell responses with precision rather than through generalized depletion (8).

### 2.4 Regulatory T cells and loss of immune tolerance

Regulatory T cells, defined by FOXP3 and CD25 expression, are critical mediators of immune tolerance. Human studies have identified alterations in Treg frequency, phenotype, and suppressive function in T1D, although findings vary across cohorts and disease stages (14, 15).

Recent single-cell profiling of peripheral blood from individuals with new-onset T1D and those undergoing partial remission identified dynamic changes in TIGIT+CCR7− Tregs and suggested functional crosstalk between these Tregs and CD226+CD8+ T cells, providing contemporary human evidence that regulatory T-cell states vary across disease phases (51).

Treg dysfunction permits uncontrolled proliferation of autoreactive effector T cells. Under inflammatory conditions, Tregs may also lose lineage stability, further compromising their suppressive function (14, 15).

Therapeutic adoptive transfer of ex vivo–expanded autologous Tregs has been shown to be safe and to have modest efficacy in preliminary clinical studies (14, 52). Newer gene-editing strategies are being used to engineer antigen-specific Tregs with improved durability, specificity, and tolerance-inducing capacity (53, 54).

### 2.5 Innate–adaptive immune crosstalk in type 1 diabetes

#### 2.5.1 Mechanistic feed-forward loop linking β-cell stress to adaptive autoimmunity

Pancreatic β-cells are increasingly recognized as active participants in T1D pathogenesis rather than passive targets of autoreactive lymphocytes. Viral infection, endoplasmic reticulum stress, oxidative stress, and inflammatory cytokines can induce β-cell danger signaling, type I interferon responses, chemokine production, and antigen release. These signals recruit and activate dendritic cells and macrophages, which process β-cell antigens and prime autoreactive CD4+ and CD8+ T cells through antigen presentation, costimulation, and inflammatory cytokines (37, 55).

This interaction becomes self-reinforcing as type I interferons and T-cell-derived IFN-γ activate JAK–STAT signaling and increase β-cell HLA class I expression, enhancing recognition by cytotoxic CD8+ T cells. Continued β-cell injury releases additional native and stress-modified antigens, promoting antigenic and epitope spreading and broadening autoreactive T-cell responses. Persistent inflammation can simultaneously impair Treg stability and suppressive function, allowing pathogenic responses to escape regulatory control. Thus, β-cell stress, innate immune activation, adaptive T-cell responses, and regulatory failure form a feed-forward circuit that progressively amplifies β-cell autoimmunity (15, 55, 56).

#### 2.5.2 Cellular contributors to innate–adaptive crosstalk

T cells do not act alone in T1D. Dynamic interactions with innate immune cells closely shape their activation, expansion, and effector functions, and this bidirectional crosstalk contributes substantially to β-cell autoimmunity. Dendritic cells are central to the early priming of autoreactive T cells by presenting β-cell antigens in pancreatic lymph nodes, thereby linking local tissue injury to adaptive immune activation. Distinct dendritic cell subsets may further influence whether immune responses become pathogenic or tolerogenic (9, 57).

Notably, conventional DCs (cDCs) are essential for pathogenicity, as CD11b^+^CD11c^+^ myeloid DCs provide critical antigenic stimulation to naïve autoreactive CD4^+^ T cells in pancreatic lymph nodes, and their ablation completely abrogates insulitis and diabetes. In contrast, plasmacytoid DCs (pDCs) exert countervailing regulatory actions; their loss accelerates insulitis, whereas their restoration attenuates islet inflammation via localized IDO-dependent regulatory mechanisms in the target tissue (58).

Macrophages contribute to disease propagation by producing inflammatory mediators such as IL-1β, TNF-α, and IL-6, which amplify local tissue inflammation, increase β-cell stress, and promote activation of autoreactive T cells (33).

Thornley et al. demonstrated that islet-resident macrophages possess an immunoregulatory phenotype capable of inducing FoxP3^+^ Tregs *in vitro*; however, this immunoregulatory phenotype, particularly CD39 expression, is progressively diminished as T1D approaches in NOD mice, coinciding with a disproportionate increase in immunostimulatory islet-resident dendritic cells relative to immunoregulatory macrophages within the islet niche (59).

In parallel, NK cells may contribute to early islet inflammation and enhance immune activation by secreting cytokines and interacting with antigen-presenting cells. These innate signals help shape the differentiation of CD4^+^ T cell subsets, support CD8^+^ cytotoxicity, and weaken regulatory immune control (34).

Beyond these classical innate cells, MAIT cells have emerged as important modulators of islet autoimmunity. Human studies have identified altered circulating MAIT-cell frequency, phenotype, and function in both pediatric and adult T1D, including activated phenotypes near disease onset and more exhausted functional profiles in long-standing disease (60, 61). However, direct pancreatic involvement in humans remains less certain, and mechanistic evidence for protective or pathogenic MAIT-cell functions derives largely from experimental models. MAIT cells, which recognize microbial riboflavin metabolites presented by MR1, exhibit tissue-dependent functional duality: in the intestinal mucosa they maintain barrier integrity through IL-17A and IL-22 production, but upon accumulation in the inflamed pancreas they produce granzyme B and IFN-γ that may contribute to β-cell death, though their net effect is protective against T1D. Circulating MAIT cell alterations are detectable before clinical onset, suggesting potential utility as biomarkers for disease progression (60).

In the NOD mouse model, γδ T cells have been shown to contribute to autoimmune diabetes, with CD27^-^CD44^hi^ γδ T cells pre-programmed to secrete IL-17 upon activation and CD27^+^CD44^hi^ γδ T cells pre-programmed to secrete IFN-γ upon activation. Both CD27^-^CD44^hi^ and CD27^+^CD44^lo^; subsets infiltrate islets of pre-diabetic NOD mice. CD27^-^ γδ T cells potently accelerate disease by cooperating with αβ T cells to transfer T1D in an IL-17–dependent manner, as antibody-mediated blockade of IL-17 prevented T1D transfer. Moreover, introgression of genetic Tcrd deficiency onto the NOD background provided robust T1D protection in a gene dose-dependent fashion, supporting a non-redundant, pathogenic role of γδ T cells in this model (62).

iNKT cells, which recognize lipid antigens presented by CD1d, show numerical and functional defects in NOD mice and humans, though human data remain scarce and contradictory. Their pharmacological activation with α-galactosylceramide prevents T1D in NOD mice through an IL-4–dependent mechanism. However, not all iNKT cell subsets are protective: IL-17–producing iNKT17 cells, whose intestinal expansion is facilitated by the gut microbiota and positively correlates with Bacteroidales abundance, can migrate from gut mucosa to pancreatic lymph nodes and pancreas, thereby promoting T1D pathogenesis. This highlights the context-dependent nature of iNKT cell contributions to T1D (57). Innate lymphoid cells (ILCs) add another layer of regulation: ILC3s maintain intestinal barrier integrity through IL-22 and antimicrobial peptide secretion, secrete IL-17 and GM-CSF to prevent immune cell activation by pathogenic microorganisms, and promote the stability and proliferation of intestinal Tregs via IL-2 secretion, enabling their migration to pancreatic lymph nodes where they regulate the autoimmune response. ILC3s also specifically regulate FFAR2, mediating IL-22 production without producing IFN-γ, which can reduce the autoimmune response against islet β cells. In intestinal biopsies of T1D patients, a notable rise in ILC1s and a decline in IL-2 and IL-22–producing ILC3s have been observed, with the inflammatory IFN-γ–producing environment of gut-associated lymphoid tissue transforming ILC3s into ILC1s, thereby impairing Treg function and promoting T1D onset (63).

Importantly, this relationship is bidirectional. Activated T cells further modulate innate immune-cell behavior through cytokine release and local inflammatory signaling, reinforcing a pathogenic loop within the islet microenvironment.

Such cellular cooperation may help explain why single-pathway interventions often show limited durability in clinical trials. A deeper understanding of innate–adaptive immune cooperation is therefore essential for developing more precise strategies to restore immune tolerance in T1D (22, 34). The integrated cellular and molecular pathways underlying T cell–mediated β-cell destruction and innate-adaptive immune crosstalk are summarized in **Figure 2**. The immunological characteristics, functional roles, and clinical relevance of major T cell populations involved in β-cell autoimmunity are summarized in **Table 1**.

**Figure 2:**
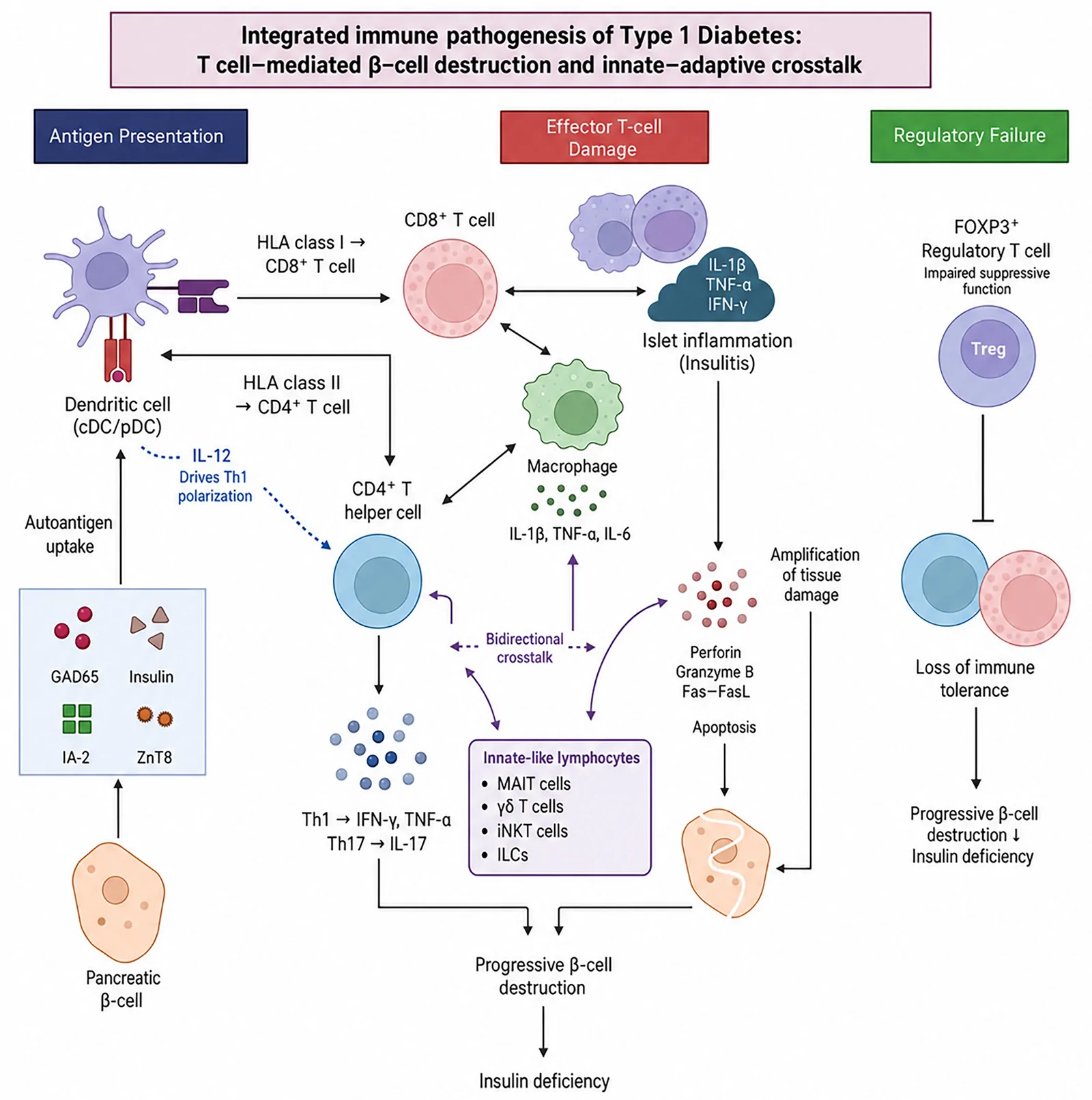
Integrated immune pathogenesis of type 1 diabetes: T cell–mediated β-cell destruction and innate–adaptive crosstalk. This schematic illustrates how coordinated interactions between innate and adaptive immune cells drive progressive β-cell destruction in type 1 diabetes. β-cell autoantigens, including insulin, GAD65, IA-2, and ZnT8, are captured by dendritic cells (cDC/pDC), which present processed peptides to autoreactive T cells through HLA class I and class II pathways. Dendritic cells further shape adaptive immunity by producing IL-12, which drives Th1 polarization of CD4^+^ T cells. HLA class I presentation activates CD8^+^ cytotoxic T cells, which directly injure β-cells through perforin, granzyme B, and Fas–FasL-mediated apoptosis. HLA class II presentation activates CD4^+^ helper T cells, which promote Th1- and Th17-associated inflammatory responses through cytokines such as IFN-γ, TNF-α, and IL-17. Macrophages amplify islet inflammation, or insulitis, by releasing IL-1β, TNF-α, and IL-6, which increase β-cell stress and further activate autoreactive T cells. Innate-like lymphocytes, including MAIT cells, γδ T cells, iNKT cells, and innate lymphoid cells, contribute to the inflammatory milieu through cytokine production and direct interactions with both innate and adaptive immune populations. Bidirectional crosstalk between activated T cells and innate immune cells reinforces a self-sustaining pathogenic loop within the islet microenvironment. Impaired FOXP3^+^ regulatory T cell function further weakens immune tolerance, allowing autoreactive T cell responses to persist. Together, cytotoxic injury, innate-driven inflammation, regulatory failure, and innate–adaptive immune amplification lead to progressive β-cell loss and insulin deficiency. Created with BioRender.com.

**Table 1:** Immunological characteristics of T cell populations driving β-cell autoimmunity in type 1 diabetes.

T cell population	Immunological identity	Functional behavior in pancreatic islets	Contribution to disease evolution	Relevance for monitoring or intervention	Ref.
CD4^+^ type 1 helper cells	T-bet–dominant polarization	Secrete pro-inflammatory mediators that activate macrophages and support cytotoxic lymphocytes	Promote an inflammatory microenvironment and β-cell stress	Linked to aggressive disease course	(9, 64)
CD4^+^ IL-17–producing cells	RORγt-driven differentiation	Recruit innate immune cells and sustain tissue inflammation	Promote chronic insulitis and amplify immune damage	Investigated as inflammatory activity indicators	(44, 64)
CD8^+^ effector T lymphocytes	Antigen-specific cytotoxic phenotype	Recognize β-cell peptides presented via HLA class I and induce apoptosis	Primary mediators of direct β-cell destruction	Central therapeutic target in T cell–directed trials	(13, 48)
Regulatory T cells (Tregs)	FOXP3-expressing suppressor cells	Suppress autoreactive T cell expansion and modulate cytokine balance	Functional impairment contributes to tolerance breakdown	Basis of adoptive Treg and engineered tolerance therapies	(14, 54)
Antigen-experienced memory T cells	Long-lived antigen-experienced T-cell populations	Retain reactivity to islet antigens and may persist over time	May contribute to persistence of autoreactive immune memory	Potential marker of persistent autoreactivity and response to immune modulation	(11, 27, 65)

## 3 Translational insights: from bench to clinic

### 3.1 T cell biomarkers in type 1 diabetes

The translation of T-cell biology into clinical practice increasingly depends on biomarkers that can define disease stage, monitor immune activity, and support patient stratification (8, 64). Autoantigen-reactive T cells recognizing β-cell antigens such as insulin, GAD65, and IA-2 can be identified in peripheral blood, although their low frequency has historically limited routine detection. Advances in peptide–MHC multimer assays, single-cell profiling, and paired TCR sequencing now permit higher-resolution characterization of antigen-specific T-cell populations (11, 26). Circulating cytokine profiles may provide complementary information on immune activation, although individual cytokines are unlikely to serve as sufficiently specific stand-alone biomarkers. Autoantibody profiling therefore remains the best-established approach for staging presymptomatic T1D, with the presence of multiple islet autoantibodies identifying individuals at high risk of progression to clinical disease (64, 66, 67). Beyond autoantibodies, high-resolution T-cell profiling and integrated immune signatures may complement established staging approaches and help identify biomarkers of disease activity and therapeutic response (11, 22, 64).

### 3.2 HLA typing and autoreactive T cell repertoires

HLA class II types, particularly HLA-DR3 and HLA-DR4, are among the most important genetic risk factors for T1D (18–20). More specifically, the primary risk factor for β-cell autoimmunity is genetic, mainly occurring in individuals with either the HLA-DR3-DQ2 or HLA-DR4-DQ8 haplotypes, or both, which together account for nearly 90% of children diagnosed with type 1 diabetes in Scandinavia (16).

These molecules determine how antigens are presented to CD4^+^ T cells and thereby shape the autoreactive T cell repertoire. Allele-specific presentation of insulin-derived peptides may favor priming of autoreactive T cells (8, 16, 20).

HLA-DQ2 and HLA-DQ8 are associated with distinct autoimmune initiation pathways: children with the DR4-DQ8 haplotype predominantly develop insulin autoantibodies (IAA) as the first-appearing autoantibody, whereas those with the DR3-DQ2 haplotype primarily develop GAD autoantibodies (GADA) (16).

Furthermore, individuals heterozygous for HLA-DQ2/8 have a synergistically increased risk compared with HLA-DQ2 or HLA-DQ8 homozygous subjects, which may result from the presence of a transdimer formed between the α-chain of HLA-DQ2 (DQA105:01) and the β-chain of HLA-DQ8 (DQB103:02), termed HLA-DQ8trans, which accommodates a unique peptide binding repertoire distinct from those of HLA-DQ2 and HLA-DQ8, consequently resulting in the presentation of a diversity of autoantigen-derived epitopes and induction of a broader repertoire of autoreactive CD4^+^ T cells (68).

Recent single-cell and paired TCR-sequencing approaches have enabled high-resolution characterization of autoreactive T-cell clonotypes and their functional states in T1D (11).

Using HLA-I peptidomics on HLA-A02:01-expressing β-cells, conventional and neo-antigenic epitopes from insulin granule proteins such as SCG5, PCSK2, UCN3, and ISL1 were naturally processed and presented; circulating naive CD8+ T cells recognizing these epitopes were detected at similar frequencies in both T1D patients and healthy donors, yet antigen-experienced CD8+ T cells reactive to IAPP, ISL1, and UCN3 peptides selectively accumulated in the pancreas of T1D patients, indicating that pancreatic inflammatory context and HLA-I upregulation by cytokines—rather than the mere existence of autoreactive repertoires—determine the transition from benign autoreactivity to pathogenic β-cell destruction (69).

These advances may support future precision-immunology approaches in which TCR signatures contribute to patient stratification and therapeutic-response monitoring (11, 26).

Importantly, HLA-associated autoreactive T cell repertoires are shaped not only by adaptive immune priming but also by the quality and context of antigen presentation by innate immune cells, particularly dendritic cell subsets in pancreatic lymph nodes (20). This underscores the interdependence of genetic risk and innate immune function in T1D pathogenesis, as MHC/HLA risk alleles and non-HLA susceptibility loci influence dendritic-cell phenotype, trafficking to pancreas-draining lymph nodes, and the ability to prime or tolerize autoreactive T cells (20, 57).

### 3.3 Translational insights from animal models

The NOD mouse has been foundational for T1D research because it reproduces many features of human disease (46, 70). NOD studies have illuminated mechanisms of CD4^+^ and CD8^+^ T cell–mediated β-cell destruction, tolerance failure, and Treg dysfunction. These models have also served as important preclinical platforms for evaluating immune-modulatory and tolerance-restoring strategies before clinical translation (14, 70).

Despite their major contribution to T1D research, NOD mice do not fully reproduce the heterogeneity or temporal course of human disease. NOD mice are genetically inbred, display strain- and sex-dependent diabetes penetrance, and develop more prominent insulitis than is typically observed in human pancreatic tissue. Differences in MHC/HLA biology, autoantigen recognition, microbiota, and immune-cell composition further limit direct extrapolation to patients. An additional challenge is therapeutic timing: many interventions that successfully prevent diabetes in NOD mice are initiated during very early preclinical disease, whereas human trials often enroll individuals at later disease stages when substantial β-cell loss has already occurred. Consequently, therapeutic efficacy in NOD mice should be interpreted as mechanistic or proof-of-concept evidence rather than direct evidence of likely clinical efficacy, and should increasingly be complemented by human tissue studies, longitudinal immune profiling, and humanized experimental systems (70, 71).

Single-cell and systems-immunology approaches have substantially expanded the translational toolkit for T1D. Single-cell transcriptomics and paired TCR sequencing enable high-resolution characterization of autoreactive T-cell populations, including rare clonotypes and distinct functional states (11). More recently, integration of single-cell multi-omic and spatial profiling of human pancreatic tissue has provided tissue-level insight into cell-type-specific regulatory programs and immune interactions across stages of T1D progression (21). Together, these approaches complement established autoantibody and metabolic staging frameworks and may facilitate the identification of biomarkers associated with disease progression and therapeutic response (64).

### 3.4 Lessons from clinical trials

Clinical trials of T cell–directed therapies have provided crucial information over several decades, despite modest therapeutic effects overall. The ability of teplizumab to delay progression to clinical T1D in at-risk individuals provides proof of concept that targeted T-cell modulation can alter the natural history of disease (25, 26). Other immune-directed interventions, including alefacept and abatacept, have demonstrated partial or transient preservation of β-cell function without durable remission, highlighting the importance of disease stage, timing of intervention, and patient selection (72–74).

Collectively, these trials demonstrate that therapeutic response in T1D is strongly influenced by disease stage, residual β-cell function, and immunological heterogeneity. Durable benefit will likely require interventions delivered at appropriate stages of disease and guided by biomarkers that identify individuals most likely to respond (25, 64, 74).

These findings support a shift toward earlier, biomarker-informed intervention and more individualized trial design. Future studies should incorporate disease stage, genetic and immune heterogeneity, and treatment-response biomarkers when selecting participants and evaluating therapeutic efficacy. Combination strategies may ultimately be required to achieve more durable immune tolerance while preserving β-cell function (25, 64, 70).

## 4 T cell–targeted therapies in type 1 diabetes

### 4.1 First-generation broad immunomodulation

Early attempts at T cell–directed therapy relied on broad immunosuppression, particularly cyclosporine. Early clinical trials demonstrated that immune modulation could temporarily preserve residual β-cell function in patients with recent-onset T1D (75, 76). However, systemic toxicity and the lack of durable benefit after treatment discontinuation limited the long-term clinical use of broad immunosuppression (74, 77, 78). These limitations shifted the field toward more selective immunotherapies designed to modulate autoreactive immune responses while preserving protective immunity. The next generation of therapies used monoclonal antibodies directed against T cell surface molecules.

### 4.2 Surface-directed monoclonal antibodies

#### 4.2.1 Anti-CD3 therapy

Anti-CD3 monoclonal antibodies that are non-FcR-binding (such as teplizumab) deliver a partial TCR/CD3 signal characterized by CD3ζ phosphorylation and ZAP-70 association but reduced ZAP-70 phosphorylation, impaired PLCγ1 activation, and absent Ca²^+^ flux, leading to clonal anergy rather than full activation (79). The ITAM-containing CD3 chains (ζ, ϵ, γ, δ) are phosphorylated after TCR–CD3 engagement, recruiting ZAP-70 and activating downstream pathways including PLCγ1 and Ras/PI3K, which drive T cell activation (79, 80).

Stimulation of T cells with immobilized or nonmitogenic anti-CD3 in the absence of costimulation induces long-lasting functional unresponsiveness (anergy) with defective IL-2 production, despite preserved TCR expression; this anergy requires partial activation and can occur independently of B7/CD28 costimulation (79, 81–83).

Teplizumab is a humanized, FcR-nonbinding anti-CD3 mAb that binds the CD3ϵ chain with reduced Fc-receptor engagement and cytokine release compared with OKT3 (25, 84). Mechanistically, it delivers partial agonistic CD3/TCR signaling that promotes deactivation or exhaustion of autoreactive CD8^+^ T cells and induces regulatory phenotypes, rather than wholesale T cell depletion (25, 84, 85). Teplizumab treatment in T1D is associated with expansion of regulatory or partially exhausted T cell subsets, including PD-1^+^FoxP3^+^ CD4^+^ Tregs and exhausted CD8^+^ cells, and with increased regulatory/exhaustion gene signatures and reduced TCR-activation signatures over months after therapy, consistent with operational tolerance (25, 27, 85).

The clinical effects of teplizumab should be interpreted according to disease stage. In stage 2 T1D, characterized by islet autoimmunity and dysglycemia without overt clinical diabetes, teplizumab delays progression to stage 3 disease, as demonstrated in the TrialNet TN-10 prevention trial (25, 26).

In newly diagnosed stage 3 T1D, when substantial residual β-cell function remains, the phase 3 PROTECT trial demonstrated significantly greater preservation of stimulated C-peptide at week 78 in children and adolescents treated with teplizumab, although key secondary clinical outcomes did not differ significantly from placebo (86). In 2026, this evidence supported FDA accelerated approval of teplizumab for delaying the decline of endogenous insulin production in patients aged 8–17 years with recently diagnosed stage 3 T1D. In contrast, no approved disease-modifying therapy currently restores or preserves β-cell function in individuals with longstanding established stage 3 T1D, emphasizing the importance of disease stage and residual β-cell mass when considering immune intervention (87).

#### 4.2.2 Anti-CD2 therapy

Anti-CD2 therapy with alefacept has been evaluated in new-onset T1D through the T1DAL phase 2 trial. Although the primary 2-hour C-peptide endpoint was not met at 12 months, alefacept was associated with preservation of β-cell function on secondary measures, reduced insulin requirements, and fewer hypoglycemic events, with evidence of sustained C-peptide preservation at 24 months. Nevertheless, alefacept has not progressed to routine clinical use, and durable disease remission has not been established (72, 74).

#### 4.2.3 Anti-CD20 therapy

Anti-CD20 therapy with rituximab depletes B cells and may indirectly modulate autoreactive T-cell responses by reducing B-cell-mediated antigen presentation. In recent-onset T1D, rituximab produced transient preservation of β-cell function and reduced insulin requirements, but it did not establish durable disease remission (74, 88).

#### 4.2.4 CTLA-4–Ig and co-stimulatory blockade

Abatacept (CTLA-4–Ig) blocks CD80/CD86-dependent T-cell costimulation and thereby reduces CD28-mediated T-cell activation. Full T-cell activation requires both TCR recognition of peptide–MHC and a costimulatory signal, and interruption of the CD28–B7 pathway can limit effector T-cell responses (89, 90). In T1D, abatacept has been shown to alter memory CD4+ T-cell and T follicular helper-cell compartments, with more recent immunophenotyping also demonstrating effects on conventional and regulatory T-cell subsets (73, 91). Clinical trials in recent-onset T1D have shown that abatacept can slow the decline of stimulated C-peptide and partially preserve β-cell function, although it does not induce durable remission (74, 92). Recent studies further suggest that baseline immune phenotypes may help identify individuals more likely to respond to abatacept, supporting a more biomarker-informed approach to costimulation blockade (73).

### 4.3 Antigen-specific immunotherapy

Antigen-specific immunotherapy aims to re-establish immune tolerance toward defined β-cell autoantigens while largely sparing systemic immunity, in contrast to broad immunosuppression (23). Selected β-cell antigens are delivered in a tolerogenic manner to delete, inactivate, or reprogram autoreactive T cells without compromising global host defense (23, 93).

Antigen-specific approaches deliver disease-relevant self-antigens under tolerogenic conditions with the aim of reducing pathogenic responses and promoting regulatory immunity (23, 93). Approaches include whole proteins, peptides, and nucleic-acid-encoded epitopes designed to induce T cell anergy, exhaustion, or regulatory T cells rather than effector responses (23, 94).

Key β-cell autoantigens targeted include insulin/proinsulin, GAD65, and IA-2, which are central to human and NOD mouse islet autoimmunity and have been used in peptide, protein, and DNA vaccine formats (94, 95).

Importantly, targeting a single autoantigen may be insufficient given the breadth of the autoreactive response, as there is no “one-antigen-fits-all” option. Precision medicine approaches using multiple disease-relevant epitopes tailored to the subject’s MHC haplotype offer a more focused way of targeting autoreactive T cells. Postigo-Fernandez et al. demonstrated that a DNA vaccine platform (Endotope) encoding multiple MHC class I and II–restricted epitopes, including neoepitopes and mimotopes, effectively prevented diabetes in NOD mice with efficacy comparable to Proinsulin pDNA in stage 1 disease, through induction of Foxp3+ regulatory T cells by at least one epitope, with the secreted form of the polypeptide, intradermal delivery, and sustained long-term weekly dosing being critical for achieving durable protection that persists after treatment discontinuation (94). In a human study, Liu et al. showed that intradermal administration of six HLA-DRB1*0401-selective β-cell peptides maintained stimulated C-peptide at ≥100% of baseline in half of treated patients with recent-onset T1D, accompanied by a dose-dependent increase in Treg expression of the transcription factor FOXP3 (96).

GAD65-based immunotherapy (GAD-alum) can modestly preserve C-peptide in subsets of newly diagnosed patients, especially those with HLA-DR3-DQ2 and immune deviation toward Th2/Treg phenotypes. Still, overall clinical efficacy has been variable and generally modest (97, 98).

DNA-vaccine platforms encoding proinsulin or multiple disease-relevant epitopes have shown tolerogenic activity in preclinical T1D models (95). More recently, mRNA-based tolerogenic approaches have also shown the ability to prevent or delay diabetes and modulate immune responses in NOD mice (99, 100).

A major hurdle remains inducing sufficiently durable, antigen-specific immune reprogramming in individuals with established autoimmune memory, as current human studies have demonstrated immune modulation but limited evidence of sustained disease reversal (23, 96).

### 4.4 Cell-based therapies

#### 4.4.1 Regulatory T cell therapies

Regulatory T cells (Tregs) are a specialized CD4+ T-cell lineage characterized by expression of the transcription factor FOXP3, which is central to their suppressive identity and function (15, 101). Tregs maintain peripheral immune tolerance through multiple, partially redundant mechanisms: (i) direct cell-contact-dependent suppression and modulation of antigen-presenting cells through molecules such as CTLA-4 and LAG-3, which reduce costimulation (15, 101); (ii) secretion of immunosuppressive cytokines, including IL-10, TGF-β, and IL-35, which dampen effector T cell activation and shape tolerogenic APCs; and (iii) metabolic disruption of effector T cells through high-affinity IL-2 receptor (CD25)-mediated IL-2 consumption and other metabolic pathways (15, 101). In T1D and other autoimmune settings, Tregs can be numerically or functionally deficient, with FOXP3 instability and pro-inflammatory polarization contributing to loss of tolerance (14, 15).

Adoptive transfer of ex vivo–expanded autologous Tregs aims to restore this suppressive balance. Early clinical studies of ex vivo–expanded autologous polyclonal Tregs in recent-onset T1D have demonstrated acceptable safety and persistence of infused cells, supporting the feasibility of Treg-based cellular therapy (14, 52). Nevertheless, the clinical efficacy and durability of polyclonal Treg therapy remain uncertain, motivating the development of approaches with greater antigen specificity, stability, and persistence (14, 53).

Next-generation engineered Treg products seek to enhance stability, antigen specificity, and persistence through genetic engineering approaches, including introduction of disease-relevant TCRs or chimeric antigen receptors (CARs) (53, 54). CAR-Tregs targeting β-cell antigens such as insulin or GAD65, or MHC–peptide complexes containing insulin epitopes, show potent, antigen-restricted suppression and prevention or reversal of diabetes in preclinical T1D models, supporting their potential to deliver focused islet-site immunoregulation while limiting systemic immunosuppression (54, 102).

#### 4.4.2 Engineered regulatory T-cell approaches

Engineered Treg strategies aim to overcome the limited antigen specificity, persistence, and functional stability of conventional polyclonal Treg therapy. Approaches include CAR-Tregs, TCR-engineered Tregs, and genetic programs designed to stabilize the regulatory phenotype. GNTI-122 provides a recent example in T1D: autologous CD4+ T cells are engineered to stably express FOXP3 together with an islet antigen–specific TCR recognizing an IGRP-derived peptide. The platform also incorporates a chemically inducible signaling complex that provides IL-2–like STAT5 signaling, addressing the limited IL-2 availability that can restrict Treg survival and expansion in the pancreatic inflammatory environment. Preclinical studies demonstrated antigen-dependent suppression of islet-reactive effector T cells, while a murine surrogate trafficked to pancreatic tissue and reduced diabetes progression (54, 103).

Despite this promise, several barriers remain before engineered Tregs can be broadly translated to T1D. Therapeutic cells must efficiently traffic to pancreatic islets and draining lymph nodes, maintain stable FOXP3 expression and suppressive identity in an inflammatory environment, and receive sufficient survival and IL-2–dependent signals without acquiring effector-like phenotypes. Antigen specificity must also be carefully optimized because excessive or bystander suppression could impair protective immune responses. In addition, genome editing introduces safety concerns related to off-target modification, genomic integrity, and long-term behavior of engineered cells, while manufacturing complexity, product consistency, persistence, and scalability remain important translational challenges (53, 54).

### 4.5 Immune checkpoint modulation in T1D

Immune checkpoint pathways provide important mechanistic insight into the maintenance of peripheral tolerance in T1D. In oncology, blockade of PD-1/PD-L1 or CTLA-4 can precipitate insulin-deficient autoimmune diabetes as an immune-related adverse event, underscoring the role of inhibitory checkpoint pathways in restraining autoreactive T-cell responses (104, 105). Conversely, therapeutic enhancement rather than blockade of inhibitory checkpoint signaling has been proposed as a strategy to reinforce immune tolerance in autoimmune disease, although such approaches remain experimental. PD-1/PD-L1 signaling contributes to the control of autoreactive T-cell activation, whereas CTLA-4-associated mechanisms support regulation of T-cell responses and Treg-mediated suppression (15, 106, 107).

### 4.6 Combination immunotherapeutic strategies

Durable remission in T1D has rarely been achieved with a single immune intervention, motivating increasing interest in rational combination strategies that target complementary components of the autoimmune response. Potential approaches include combining T-cell–directed therapy with antigen-specific tolerance induction, regulatory-cell therapy, or interventions that modify the inflammatory immune environment. Such strategies will likely need to account for disease stage, residual β-cell function, and immune heterogeneity to optimize efficacy while limiting systemic immunosuppression (25, 70, 74).

Autoreactive T cells operate within a broader immune network in which dendritic cells and macrophages shape antigen presentation and inflammatory signaling, while activated T cells reciprocally influence innate immune activity. Accordingly, coordinated modulation of adaptive T-cell responses together with tolerogenic or innate immune pathways may provide a more comprehensive approach to restoring immune homeostasis. However, the optimal combinations, treatment sequence, and timing remain to be established in clinical studies (33, 34, 57).

An overview of current and investigational T cell–targeted therapeutic strategies is presented in **Figure 3**. The evolution, mechanisms, and clinical outcomes of T cell-targeted therapeutic approaches are summarized in **Table 2**.

**Figure 3:**
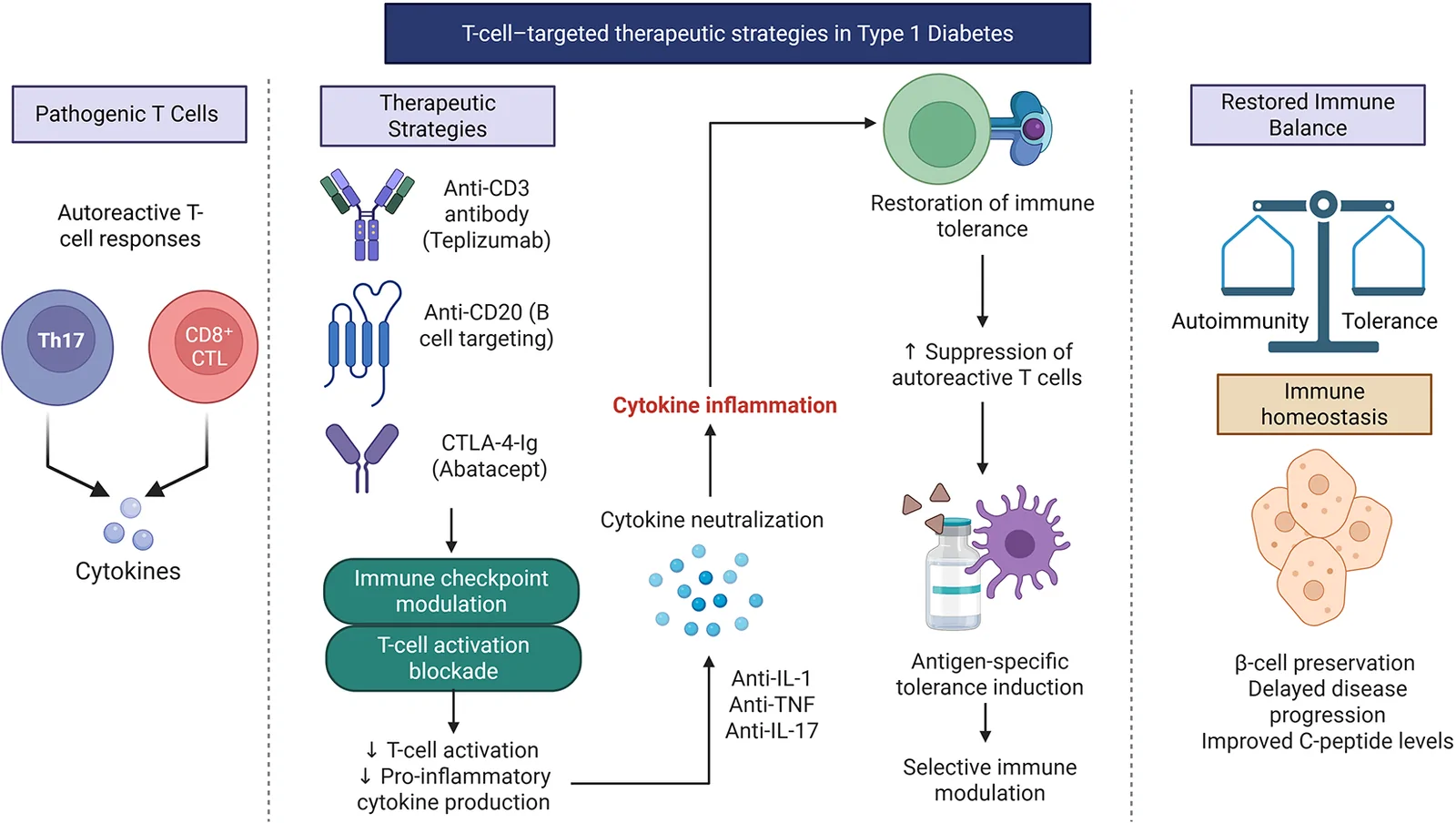
Current and emerging T cell–targeted therapeutic strategies in type 1 diabetes. The figure depicts the major immunotherapeutic approaches designed to interrupt autoreactive T cell–mediated β-cell destruction in T1D, organized from pathogenic immune drivers to downstream clinical outcomes. (Left) In T1D, autoreactive Th17 cells and CD8^+^ CTLs constitute the principal pathogenic effectors, secreting pro-inflammatory cytokines that amplify insulitis and promote β-cell destruction. (Center-left) Three classes of surface-directed monoclonal antibody therapies are shown: anti-CD3 (teplizumab), which induces T cell anergy and promotes Treg expansion; anti-CD20, which depletes B cells and indirectly reduces antigen presentation to autoreactive T cells; and CTLA-4–Ig (abatacept), which competitively blocks the CD28–B7 co-stimulatory interaction. These strategies converge on immune checkpoint modulation and T cell activation blockade, collectively reducing T cell activation and pro-inflammatory cytokine production. (Center) A parallel strategy employs cytokine neutralization with anti-IL-1, anti-TNF, and anti-IL-17 biologics to suppress the inflammatory milieu that sustains autoreactive T cell activity. (Center-right) T cell-directed and cytokine-targeted interventions together drive restoration of immune tolerance, characterized by enhanced suppression of autoreactive T cells. Antigen-specific immunotherapy approaches—including peptide vaccines, tolerogenic dendritic cells, and adoptive Treg transfer—further reinforce selective immune modulation. (Right) Successful intervention restores immune homeostasis by rebalancing autoimmunity toward tolerance, yielding key clinical outcomes: β-cell preservation, delayed disease progression, and improved C-peptide levels. Abbreviations: T1D, type 1 diabetes; CTL, cytotoxic T lymphocyte; Th17, T helper 17 cell; Treg, regulatory T cell; CTLA-4, cytotoxic T-lymphocyte–associated protein 4; IL, interleukin; TNF, tumor necrosis factor; CD, cluster of differentiation; B7, B7 ligands (CD80/CD86). Created with BioRender.com.

**Table 2:** Evolution of T cell-focused therapeutic approaches in type 1 diabetes: mechanistic intent and clinical reality.

Therapeutic approach	Immunological objective	Observed clinical effect	Current development status	Key translational barrier	Ref.
Non-selective immunosuppressants (e.g., calcineurin inhibitors)	Broad attenuation of T cell activation	Temporary preservation of endogenous insulin production	Historically explored; not continued	Systemic toxicity and relapse after discontinuation	(74–76)
Anti-CD3 monoclonal antibodies (e.g., teplizumab)	Reset autoreactive T cell responsiveness and promote regulatory pathways	Stage 2: delays progression to stage 3 T1D. Newly diagnosed pediatric stage 3: preserves endogenous insulin production/C-peptide.	FDA-approved for stage 2 T1D; accelerated approval (2026) for recently diagnosed stage 3 T1D in patients aged 8–17 years.	Stage-dependent benefit, variable response, and durability; no approved disease-modifying therapy for longstanding established stage 3 T1D.	(25, 26, 86)
CD2 pathway blockade	Reduce memory T cell activation	Partial preservation of β-cell function in new-onset T1D	Phase II completed; not pursued clinically	Limited evidence of durable disease modification	(72, 74)
Co-stimulation modulators (CTLA-4–Ig)	Interrupt secondary activation signals in T cells	Slowed decline of stimulated C-peptide in recent-onset T1D	Phase II clinical evaluation	Partial efficacy without durable remission	(73, 92)
Anti-CD20 B-cell depletion (Rituximab)	Indirect reduction of antigen presentation to T cells	Transient preservation of β-cell function	Phase II	Benefit diminishes over time; no durable remission	(74, 88)
Antigen-specific immunotherapy	Re-establish tolerance toward β-cell proteins	Generally safe; immune modulation with variable metabolic benefit	Preclinical to early clinical development	Heterogeneous antigen responses and limited durability	(23, 96)
Tolerogenic dendritic cell therapy	Promote regulatory immune circuits	Demonstrated safety and immune modulation	Early-stage trials	Scalability and long-term persistence unknown	(93, 108)
Autologous Treg infusion	Reinforce endogenous immune suppression	Demonstrated safety and persistence of infused Tregs	Early clinical development	Durability, stability, and antigen specificity	(14, 52)
Engineered Treg platforms/CAR-Tregs	Antigen-restricted immune suppression	Preclinical evidence of targeted immune regulation	Preclinical/experimental	Safety, manufacturing complexity, persistence, and scalability	(53, 54)
Immune checkpoint modulation	Reinforce inhibitory pathways that contribute to peripheral tolerance	Preclinical/mechanistic evidence	Experimental	Potential systemic immune effects and uncertain clinical efficacy	(15, 106)

### 4.7 Lessons learned and remaining challenges

Several consistent lessons have emerged from clinical studies of immune-directed therapy in T1D. First, timing is critical: interventions are more likely to preserve meaningful β-cell function when administered before extensive β-cell loss has occurred, supporting increasing emphasis on presymptomatic and early-stage disease (25, 26, 64).

Second, broad immunosuppression is limited by systemic toxicity and the inability to maintain treatment indefinitely, reinforcing the need for more selective and tolerogenic approaches. Even targeted therapies have generally produced partial or temporary preservation of β-cell function rather than permanent immune tolerance, making durability a major unresolved challenge (70, 74).

Finally, safety is particularly important when immune intervention is considered for children or asymptomatic individuals at risk of progression. Future therapeutic development will therefore require careful risk–benefit assessment together with disease-stage and biomarker-informed patient selection to maximize benefit while minimizing unnecessary immune suppression (25, 64). A detailed overview of individual T cell–targeted therapies, their mechanisms, clinical outcomes, and limitations is presented in **Table 3**. The clinical application and translational impact of these T cell–targeted therapeutic strategies are illustrated in **Figure 4**.

**Table 3:** T cell-targeted therapies in type 1 diabetes: mechanisms, clinical trial outcomes, and limitations.

Therapy	Target/mechanism	Clinical outcome	Stage of development	Limitations
Rituximab (anti-CD20)	B-cell depletion indirectly modulates autoreactive T-cell responses	Transient preservation of β-cell function and reduced insulin requirements	Phase II	Benefit diminishes over time; no durable remission
Antigen-specific immunotherapy	Induction of antigen-specific tolerance to β-cell autoantigens	Immune modulation with variable and generally modest metabolic benefit	Preclinical to early clinical development	Heterogeneous antigen responses and limited durability
Tolerogenic dendritic cells	Promote antigen-specific regulatory immune responses and suppress autoreactive T cells	Early clinical studies demonstrate safety and immune modulation	Early clinical development	Limited clinical experience; long-term efficacy and scalability remain uncertain
Adoptive Tregs	Ex vivo–expanded autologous Tregs to reinforce immune suppression	Demonstrated safety and persistence of infused cells	Early clinical development	Durability, functional stability, and antigen specificity remain challenges
Engineered Tregs/CAR-Tregs	Antigen-specific Tregs engineered with CARs/TCRs and stabilized regulatory programs	Targeted immune suppression in preclinical T1D models	Preclinical/experimental	Trafficking, Treg stability, IL-2 dependence, off-target suppression, genome-editing safety, manufacturing, and scalability
Immune checkpoint modulation	Reinforcement of inhibitory pathways involved in peripheral immune tolerance	Mechanistic and preclinical rationale; clinical efficacy in T1D remains unestablished	Experimental	Potential systemic immune effects and uncertain therapeutic window

**Figure 4:**
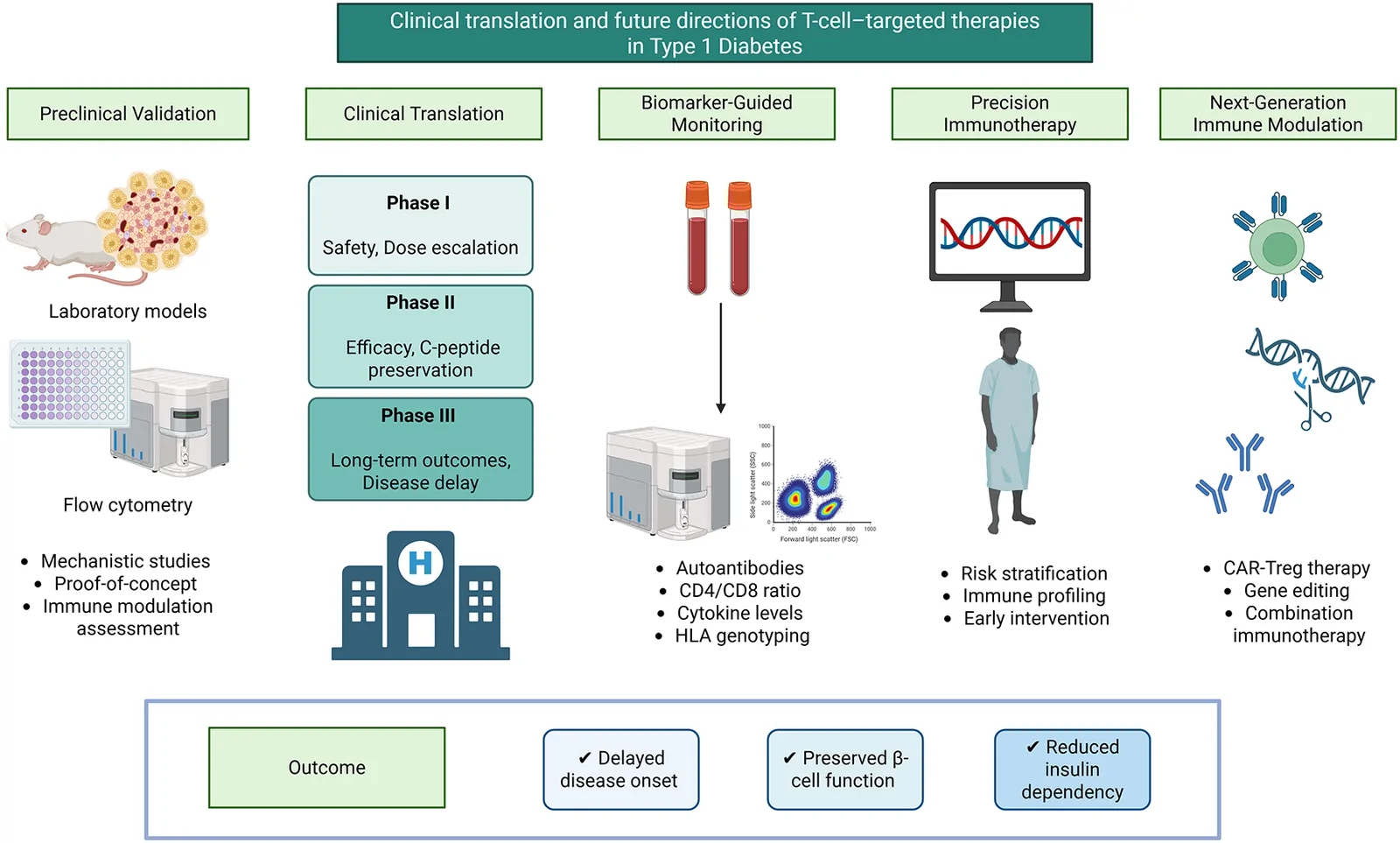
Clinical translation and future directions of T cell–targeted therapies in type 1 diabetes. This schematic outlines the translational pathway for developing and applying T cell–targeted immunotherapies in type 1 diabetes. Preclinical validation relies on laboratory models, mechanistic studies, proof-of-concept experiments, and immune-modulation assays such as flow cytometry. Promising approaches then advance through phased clinical evaluation, beginning with safety and dose-escalation studies in Phase I, followed by efficacy and C-peptide preservation in Phase II, and long-term outcomes, disease delay, and durability in Phase III. Biomarker-guided monitoring, including autoantibody profiling, CD4/CD8 ratios, cytokine measurements, HLA genotyping, and immune phenotyping, supports patient selection and treatment-response assessment. Precision immunotherapy integrates immune profiling and risk stratification to identify individuals most likely to benefit from early intervention. Next-generation strategies include CAR-Treg therapy, gene editing, and combination immunotherapy. The intended clinical outcomes include delayed disease onset, preservation of β-cell function, and reduced insulin dependency. Created with BioRender.com.

## 5 Current challenges and future perspectives

### 5.1 Disease heterogeneity in type 1 diabetes

T1D is a heterogeneous autoimmune disease characterized by substantial variation in age at onset, rate of β-cell loss, genetic susceptibility, immune phenotype, and clinical progression. Some individuals develop rapidly progressive disease during childhood, whereas others present later in life with a more gradual decline in β-cell function. This heterogeneity has important implications for disease classification, prediction, and therapeutic response (64, 70).

Genetic variation contributes substantially to this diversity, particularly through HLA-associated susceptibility and additional immune-regulatory risk loci that can influence autoreactive immune responses (20, 64). Environmental exposures, viral infections, and interactions between the intestinal immune system and microbiota may further modify disease initiation and progression, adding additional layers of biological heterogeneity (63).

### 5.2 Timing of therapeutic intervention

The timing of immune intervention is a major determinant of therapeutic benefit in T1D. Because progressive β-cell dysfunction begins before symptomatic disease, intervention during presymptomatic stages offers an opportunity to modify autoimmunity while greater endogenous β-cell function remains. The clinical efficacy of teplizumab in individuals with stage 2 T1D provides proof of concept that appropriately timed immune intervention can delay progression to stage 3 disease (25, 26, 64).

These advances have increased interest in screening strategies capable of identifying individuals before clinical diagnosis. Islet autoantibodies remain central to risk assessment and disease staging, but implementation of population-level screening raises practical questions regarding whom to screen, testing frequency, cost, follow-up, and access to preventive interventions. Screening programs must also consider the psychological and clinical implications of identifying asymptomatic individuals at elevated risk of future disease (64, 70).

Accordingly, preventive immunotherapy requires a particularly favorable safety profile because treatment may be offered to otherwise asymptomatic individuals. Future strategies will need to integrate disease stage, residual β-cell function, immune biomarkers, and individualized risk when determining the optimal timing of intervention (25, 64).

### 5.3 Durability and safety of immune modulation

Durability remains a major challenge for immune-directed therapy in T1D. Teplizumab can delay progression to stage 3 disease but does not permanently prevent T1D in all treated individuals, while abatacept and other immune-modulatory approaches have generally produced partial preservation of β-cell function rather than sustained disease remission (25, 74). Similarly, although adoptively transferred Tregs can persist after infusion, their long-term capacity to maintain clinically meaningful immune tolerance remains uncertain (14, 52).

Safety is equally important, particularly because immune interventions may be administered to children or asymptomatic individuals at risk of disease progression. Broad immunosuppression is limited by systemic toxicity and the risks associated with sustained suppression of protective immunity, whereas more targeted biologic therapies can still produce treatment-related adverse effects such as cytokine-release symptoms, lymphopenia, or other alterations in immune function (25, 74). Furthermore, the development of autoimmune diabetes following immune-checkpoint blockade illustrates the potential consequences of disrupting pathways such as PD-1/PD-L1 and CTLA-4 that normally contribute to peripheral tolerance (104, 105). Future therapies must therefore balance efficacy and durability with preservation of protective immunity while aiming to establish stable, antigen-specific immune tolerance.

### 5.4 Systems biology and multi-omics approaches

Systems-level and multi-omic approaches are increasingly refining the study of immune heterogeneity in T1D. Single-cell transcriptomics, paired TCR sequencing, proteomic profiling, and related technologies can resolve rare autoreactive T-cell populations and characterize their functional states at substantially greater resolution than conventional bulk analyses (11, 109). Integration of single-cell multi-omic analysis with spatial profiling of human pancreatic tissue has further enabled characterization of cell-type-specific regulatory programs and immune interactions within the tissue context of T1D progression (21). These approaches may help identify composite immune signatures associated with disease stage, progression, and therapeutic response, complementing established autoantibody and metabolic staging frameworks (64).

A major challenge is translating high-dimensional molecular datasets into biomarkers that are reproducible, clinically interpretable, and generalizable across populations. Future studies will require standardized analytical pipelines, longitudinal validation, and diverse study populations before multi-omic or computational signatures can be incorporated reliably into routine risk prediction or therapeutic stratification.

### 5.5 Precision and combination therapies

Precision immunotherapy in T1D will likely require matching therapeutic strategy to disease stage, residual β-cell function, and individual immune characteristics rather than applying a uniform intervention across all patients. Biomarker-informed stratification using autoantibody status, genetic risk, T-cell phenotypes, and other immune signatures may help identify individuals most likely to benefit from particular interventions (64, 70).

Combination therapy is also being explored as a means of targeting complementary components of the autoimmune response. Potential strategies include combining T-cell–directed therapy with antigen-specific tolerance induction, regulatory-cell approaches, or interventions that modify the inflammatory immune environment. However, evidence for optimal combinations, sequencing, and timing remains limited, and these approaches will require rigorous clinical validation rather than assuming that combinations are inherently superior to monotherapy (70, 74).

Therapeutic priorities may also differ across disease stages. Individuals with presymptomatic disease may benefit most from interventions designed to delay progression while substantial endogenous β-cell function remains, whereas later-stage disease may ultimately require integration of immune modulation with strategies aimed at restoring or replacing β-cell function (25, 64, 70).

### 5.6 Ethical and regulatory considerations

Preventive immunotherapy in T1D raises important ethical and practical considerations because treatment may be offered to children or other asymptomatic individuals who have not yet developed clinical disease. In this setting, the potential benefit of delaying progression must be weighed carefully against treatment-related risks, uncertainty regarding individual disease trajectory, and the psychosocial implications of identifying individuals as being at high risk for future T1D (25, 64).

These considerations reinforce the need for clear risk communication, informed decision-making, and a favorable safety profile for preventive interventions. As screening and disease-modifying therapies expand, equitable access to risk assessment, specialist follow-up, and treatment will also be important to avoid widening disparities in T1D care. Continued collaboration among investigators, clinicians, regulatory agencies, and patient communities will be essential for translating preventive immunotherapy into responsible clinical practice (64, 70).

### 5.7 Lessons from other immune-mediated diseases

Experience from other immune-mediated diseases and oncology provides useful principles for the development of T1D immunotherapy. In multiple sclerosis, the clinical efficacy of targeted immune therapies, such as anti-CD20 treatment, demonstrates that selective modulation of immune pathways can substantially alter disease activity, although the underlying biology and therapeutic requirements differ across autoimmune diseases (110). In oncology, immune-checkpoint blockade illustrates both the therapeutic potential and the risks of manipulating T-cell regulatory pathways, as disruption of peripheral tolerance can produce clinically significant immune-related adverse events, including autoimmune diabetes (104, 111).

These experiences emphasize that immune therapies cannot simply be transferred between diseases without consideration of disease-specific mechanisms, stage, and risk–benefit profiles. In T1D, advances in immune profiling, multi-omic technologies, and disease staging may increasingly support more precise selection and monitoring of immune interventions. Ultimately, successful translation will require therapies that achieve meaningful and durable preservation of β-cell function while minimizing disruption of protective immunity (64, 70).

## 6 Conclusion

T1D is increasingly understood as a heterogeneous, stage-dependent autoimmune disease in which autoreactive T cells interact with stressed β-cells and innate immune populations to sustain progressive β-cell loss. Although recent human single-cell and tissue studies have strengthened this framework, mechanistic causality still relies substantially on experimental models, emphasizing the need for continued validation in human disease. Teplizumab demonstrates that immune intervention can modify disease progression, but its benefits remain stage-dependent and do not yet establish durable immune tolerance. Future progress will therefore require better patient stratification, validated immune biomarkers, and therapies that selectively disrupt pathogenic immune circuits while preserving protective immunity. Combining longitudinal human profiling with antigen-specific and engineered regulatory approaches may ultimately enable more durable and precise β-cell preservation.

## References

[1] Atkinson MA Eisenbarth GS Michels AW . Type 1 diabetes. Lancet. (2014) 383:69–82. doi: 10.1016/s0140-6736(13)60591-7 23890997 PMC4380133

[2] Skyler JS . Prevention and reversal of type 1 diabetes--past challenges and future opportunities. Diabetes Care. (2015) 38:997–1007. doi: 10.2337/dc15-0349 25998292

[3] Thomas NJ Jones SE Weedon MN Shields BM Oram RA Hattersley AT . Frequency and phenotype of type 1 diabetes in the first six decades of life: a cross-sectional, genetically stratified survival analysis from UK Biobank. Lancet Diabetes Endocrinol. (2018) 6:122–9. doi: 10.1016/s2213-8587(17)30362-5 29199115 PMC5805861

[4] Gregory GA Robinson TIG Linklater SE Wang F Colagiuri S de Beaufort C . Global incidence, prevalence, and mortality of type 1 diabetes in 2021 with projection to 2040: a modelling study. Lancet Diabetes Endocrinol. (2022) 10:741–60. doi: 10.1016/s2213-8587(22)00218-2 36113507

[5] Lind M Svensson AM Kosiborod M Gudbjörnsdottir S Pivodic A Wedel H . Glycemic control and excess mortality in type 1 diabetes. N Engl J Med. (2014) 371:1972–82. doi: 10.1056/nejmoa1408214 25409370

[6] Livingstone SJ Looker HC Hothersall EJ Wild SH Lindsay RS Chalmers J . Risk of cardiovascular disease and total mortality in adults with type 1 diabetes: Scottish registry linkage study. PloS Med. (2012) 9:e1001321. doi: 10.1371/journal.pmed.1001321 23055834 PMC3462745

[7] Bluestone JA Herold K Eisenbarth G . Genetics, pathogenesis and clinical interventions in type 1 diabetes. Nature. (2010) 464:1293–300. doi: 10.1038/nature08933 20432533 PMC4959889

[8] Battaglia M Ahmed S Anderson MS Atkinson MA Becker D Bingley PJ . Introducing the endotype concept to address the challenge of disease heterogeneity in type 1 diabetes. Diabetes Care. (2020) 43:5–12. doi: 10.2337/dc19-0880 PMC6925574 31753960

[9] Pugliese A . Autoreactive T cells in type 1 diabetes. J Clin Invest. (2017) 127:2881–91. doi: 10.1172/jci94549 28762987 PMC5531393

[10] Rodriguez-Calvo T Christoffersson G Bender C von Herrath MG Mallone R Kent SC . Means, motive, and opportunity: Do non-islet-reactive infiltrating T cells contribute to autoimmunity in type 1 diabetes? Front Immunol. (2021) 12:2021. doi: 10.3389/fimmu.2021.683091 34220832 PMC8242234

[11] Eugster A Lorenc A Kotrulev M Kamra Y Goel M Steinberg-Bains K . Physiological and pathogenic T cell autoreactivity converge in type 1 diabetes. Nat Commun. (2024) 15:9204. doi: 10.1038/s41467-024-53255-9 39472557 PMC11522472

[12] Lieberman SM DiLorenzo TP . A comprehensive guide to antibody and T-cell responses in type 1 diabetes. Tissue Antigens. (2003) 62:359–77. doi: 10.1034/j.1399-0039.2003.00152.x 14617043

[13] Coppieters KT Dotta F Amirian N Campbell PD Kay TW Atkinson MA . Demonstration of islet-autoreactive CD8 T cells in insulitic lesions from recent onset and long-term type 1 diabetes patients. J Exp Med. (2012) 209:51–60. doi: 10.1084/jem.20111187 22213807 PMC3260877

[14] Huang Q Zhu J . Regulatory T cell-based therapy in type 1 diabetes: Latest breakthroughs and evidence. Int Immunopharmacol. (2024) 140:112724. doi: 10.1016/j.intimp.2024.112724 39098233

[15] Sumida TS Cheru NT Hafler DA . The regulation and differentiation of regulatory T cells and their dysfunction in autoimmune diseases. Nat Rev Immunol. (2024) 24:503–17. doi: 10.1038/s41577-024-00994-x 38374298 PMC11216899

[16] Todd JA . Etiology of type 1 diabetes. Immunity. (2010) 32:457–67. doi: 10.1016/j.immuni.2010.04.001 20412756

[17] van Belle TL Coppieters KT von Herrath MG . Type 1 diabetes: etiology, immunology, and therapeutic strategies. Physiol Rev. (2011) 91:79–118. doi: 10.1152/physrev.00003.2010 21248163

[18] Noble JA Erlich HA . Genetics of type 1 diabetes. Cold Spring Harb Perspect Med. (2012) 2:a007732. doi: 10.1101/cshperspect.a007732 22315720 PMC3253030

[19] Pociot F Lernmark Å . Genetic risk factors for type 1 diabetes. Lancet. (2016) 387:2331–9. doi: 10.1016/s0140-6736(16)30582-7 27302272

[20] Gootjes C Zwaginga JJ Roep BO Nikolic T . Functional impact of risk gene variants on the autoimmune responses in type 1 diabetes. Front Immunol. (2022) 13:886736. doi: 10.3389/fimmu.2022.886736 35603161 PMC9114814

[21] Melton R Jimenez S Elison W Tucciarone L Howell A Wang G . Single-cell multiome and spatial profiling reveals pancreas cell type-specific gene regulatory programs of type 1 diabetes progression. Sci Adv. (2025) 11:eady0080. doi: 10.1126/sciadv.ady0080 40929272 PMC12422192

[22] Bluestone JA Buckner JH Herold KC . Immunotherapy: Building a bridge to a cure for type 1 diabetes. Science. (2021) 373:510–6. doi: 10.1126/science.abh1654 34326232

[23] Peakman M Santamaria P . Autoantigen-specific immunotherapies for the prevention and treatment of type 1 diabetes. Cold Spring Harb Perspect Med. (2025) 15:a041598. doi: 10.1101/cshperspect.a041598 PMC12128869 39284667

[24] Sherry N Hagopian W Ludvigsson J Jain SM Wahlen J Ferry RJ Jr. . Teplizumab for treatment of type 1 diabetes (Protégé study): 1-year results from a randomised, placebo-controlled trial. Lancet. (2011) 378:487–97. doi: 10.1016/s0140-6736(11)60931-8 21719095 PMC3191495

[25] Mathieu C Sims EK Chatenoud L James EA Atkinson MA Herold KC . Toward disease-modifying therapies in type 1 diabetes: Focus on teplizumab. Diabetes Care. (2026) 49:365–74. doi: 10.2337/dci25-0066 PMC12925991 41196630

[26] Herold KC Bundy BN Long SA Bluestone JA DiMeglio LA Dufort MJ . An anti-CD3 antibody, teplizumab, in relatives at risk for type 1 diabetes. N Engl J Med. (2019) 381:603–13. doi: 10.1056/nejmoa1902226 31180194 PMC6776880

[27] Lledo-Delgado A Preston-Hurlburt P Currie S Clark P Linsley PS Long SA . Teplizumab induces persistent changes in the antigen-specific repertoire in individuals at risk for type 1 diabetes. J Clin Invest. (2024) 134:e177492. doi: 10.1172/jci177492 39137044 PMC11405034

[28] Eizirik DL Colli ML Ortis F . The role of inflammation in insulitis and beta-cell loss in type 1 diabetes. Nat Rev Endocrinol. (2009) 5:219–26. doi: 10.1038/nrendo.2009.21 19352320

[29] Warshauer JT Bluestone JA Anderson MS . New frontiers in the treatment of type 1 diabetes. Cell Metab. (2020) 31:46–61. doi: 10.1016/j.cmet.2019.11.017 31839487 PMC6986815

[30] Gitelman SE Bluestone JA . Regulatory T cell therapy for type 1 diabetes: May the force be with you. J Autoimmun. (2016) 71:78–87. doi: 10.1016/j.jaut.2016.03.011 27133597

[31] Citro A Campo F Dugnani E Piemonti L . Innate immunity mediated inflammation and beta cell function: Neighbors or enemies? Front Endocrinol (Lausanne). (2020) 11:606332. doi: 10.3389/fendo.2020.606332 PMC7897669 33628197

[32] Zirpel H Roep BO . Islet-resident dendritic cells and macrophages in type 1 diabetes: In search of Bigfoot's print. Front Endocrinol (Lausanne). (2021) 12:666795. doi: 10.3389/fendo.2021.666795 33912139 PMC8072455

[33] Cobo-Vuilleumier N Rodriguez-Fernandez S Lopez-Noriega L Lorenzo PI Franco JM Lachaud CC . LRH-1/NR5A2 targets mitochondrial dynamics to reprogram type 1 diabetes macrophages and dendritic cells into an immune tolerance phenotype. Clin Transl Med. (2024) 14:e70134. doi: 10.1002/ctm2.70134 39702941 PMC11659195

[34] Roy S Pokharel P Piganelli JD . Decoding the immune dance: Unraveling the interplay between beta cells and type 1 diabetes. Mol Metab. (2024) 88:101998. doi: 10.1016/j.molmet.2024.101998 39069156 PMC11342121

[35] Quinn LM Wong FS Narendran P . Environmental determinants of type 1 diabetes: From association to proving causality. Front Immunol. (2021) 12:737964. doi: 10.3389/fimmu.2021.737964 34659229 PMC8518604

[36] Del Chierico F Rapini N Deodati A Matteoli MC Cianfarani S Putignani L . Pathophysiology of type 1 diabetes and gut microbiota role. Int J Mol Sci. (2022) 23:14650. doi: 10.3390/ijms232314650 36498975 PMC9737253

[37] Lemos JRN Hirani K von Herrath M . Immunological and virological triggers of type 1 diabetes: Insights and implications. Front Immunol. (2023) 14:1326711. doi: 10.3389/fimmu.2023.1326711 PMC10794398 38239343

[38] Nekoua MP Alidjinou EK Hober D . Persistent coxsackievirus B infection and pathogenesis of type 1 diabetes mellitus. Nat Rev Endocrinol. (2022) 18:503–16. doi: 10.1038/s41574-022-00688-1 35650334 PMC9157043

[39] Hober D Sauter P . Pathogenesis of type 1 diabetes mellitus: Interplay between enterovirus and host. Nat Rev Endocrinol. (2010) 6:279–89. doi: 10.1038/nrendo.2010.27 20351698

[40] Blok L Hanssen N Nieuwdorp M Rampanelli E . From microbes to metabolites: Advances in gut microbiome research in type 1 diabetes. Metabolites. (2025) 15:138. doi: 10.3390/metabo15020138 39997763 PMC11857261

[41] Vatanen T Franzosa EA Schwager R Tripathi S Arthur TD Vehik K . The human gut microbiome in early-onset type 1 diabetes from the TEDDY study. Nature. (2018) 562:589–94. doi: 10.1038/s41586-018-0620-2 30356183 PMC6296767

[42] Craig ME Kim KW Isaacs SR Penno MA Hamilton-Williams EE Couper JJ . Early-life factors contributing to type 1 diabetes. Diabetologia. (2019) 62:1823–34. doi: 10.1007/s00125-019-4942-x 31451871

[43] Bending D De la Peña H Veldhoen M Phillips JM Uyttenhove C Stockinger B . Highly purified Th17 cells from BDC2.5NOD mice convert into Th1-like cells in NOD/SCID recipient mice. J Clin Invest. (2009) 119:565–73. doi: 10.1172/jci37865 19188681 PMC2648686

[44] Arif S Moore F Marks K Bouckenooghe T Dayan CM Planas R . Peripheral and islet interleukin-17 pathway activation characterizes human autoimmune diabetes and promotes cytokine-mediated β-cell death. Diabetes. (2011) 60:2112–9. doi: 10.2337/db10-1643 21659501 PMC3142078

[45] Biradar R Kalim UU Lonnberg T Junttila S Suomi T Norman SZ . Single-cell RNA-seq analysis of longitudinal CD4(+) T cell samples reveals cell-type-specific changes during early stages of type 1 diabetes. Genome Med. (2025) 17:154. doi: 10.1186/s13073-025-01574-x 41462339 PMC12751217

[46] Anderson MS Bluestone JA . The NOD mouse: a model of immune dysregulation. Annu Rev Immunol. (2005) 23:447–85. doi: 10.1146/annurev.immunol.23.021704.115643 15771578

[47] Tsai S Shameli A Santamaria P . CD8+ T cells in type 1 diabetes. Adv Immunol. (2008) 100:79–124. doi: 10.1016/s0065-2776(08)00804-3 19111164

[48] Yang K Zhang Y Ding J Li Z Zhang H Zou F . Autoimmune CD8+ T cells in type 1 diabetes: From single-cell RNA sequencing to T-cell receptor redirection. Front Endocrinol (Lausanne). (2024) 15:1377322. doi: 10.3389/fendo.2024.1377322 38800484 PMC11116783

[49] Willcox A Richardson SJ Bone AJ Foulis AK Morgan NG . Analysis of islet inflammation in human type 1 diabetes. Clin Exp Immunol. (2009) 155:173–81. doi: 10.1111/j.1365-2249.2008.03860.x PMC2675247 19128359

[50] Okamura T Kitagawa N Kitagawa N Sakai K Sumi M Kobayashi G . Single-cell analysis reveals islet autoantigen's immune activation in type 1 diabetes patients. J Clin Biochem Nutr. (2025) 76:64–84. doi: 10.3164/jcbn.24-86 39896168 PMC11782777

[51] Zhong T Li X Lei K Tang R Deng Q Love PE . TGF-beta-mediated crosstalk between TIGIT(+) Tregs and CD226(+)CD8(+) T cells in the progression and remission of type 1 diabetes. Nat Commun. (2024) 15:8894. doi: 10.1038/s41467-024-53264-8 39406740 PMC11480485

[52] Bluestone JA Buckner JH Fitch M Gitelman SE Gupta S Hellerstein MK . Type 1 diabetes immunotherapy using polyclonal regulatory T cells. Sci Transl Med. (2015) 7:315ra189. doi: 10.1126/scitranslmed.aad4134 26606968 PMC4729454

[53] Tuomela K Levings MK . Genetic engineering of regulatory T cells for treatment of autoimmune disorders including type 1 diabetes. Diabetologia. (2024) 67:611–22. doi: 10.1007/s00125-023-06076-2 38236408

[54] Uenishi GI Repic M Yam JY Landuyt A Saikumar-Lakshmi P Guo T . GNTI-122: an autologous antigen-specific engineered Treg cell therapy for type 1 diabetes. JCI Insight. (2024) 9:e171844. doi: 10.1172/jci.insight.171844 38516892 PMC11063937

[55] James EA Joglekar AV Linnemann AK Russ HA Kent SC . The beta cell-immune cell interface in type 1 diabetes (T1D). Mol Metab. (2023) 78:101809. doi: 10.1016/j.molmet.2023.101809 37734713 PMC10622886

[56] Russell MA Richardson SJ Morgan NG . The role of the interferon/JAK-STAT axis in driving islet HLA-I hyperexpression in type 1 diabetes. Front Endocrinol (Lausanne). (2023) 14:1270325. doi: 10.3389/fendo.2023.1270325 37867531 PMC10588626

[57] Khan FU Khongorzul P Raki AA Rajasekaran A Gris D Amrani A . Dendritic cells and their immunotherapeutic potential for treating type 1 diabetes. Int J Mol Sci. (2022) 23:4885. doi: 10.3390/ijms23094885 35563276 PMC9099521

[58] Saxena V Ondr JK Magnusen AF Munn DH Katz JD . The countervailing actions of myeloid and plasmacytoid dendritic cells control autoimmune diabetes in the nonobese diabetic mouse. J Immunol. (2007) 179:5041–53. doi: 10.4049/jimmunol.179.8.5041 17911589

[59] Thornley TB Agarwal KA Kyriazis P Ma L Chipashvili V Aker JE . Contrasting roles of islet resident immunoregulatory macrophages and dendritic cells in experimental autoimmune type 1 diabetes. PloS One. (2016) 11:e0150792. doi: 10.1371/journal.pone.0150792 26943809 PMC4778921

[60] Rouxel O Da Silva J Beaudoin L Nel I Tard C Cagninacci L . Cytotoxic and regulatory roles of mucosal-associated invariant T cells in type 1 diabetes. Nat Immunol. (2017) 18:1321–31. doi: 10.1038/ni.3854 28991267 PMC6025738

[61] Nel I Beaudoin L Gouda Z Rousseau C Soulard P Rouland M . MAIT cell alterations in adults with recent-onset and long-term type 1 diabetes. Diabetologia. (2021) 64:2306–21. doi: 10.1007/s00125-021-05527-y 34350463 PMC8336671

[62] Markle JG Mortin-Toth S Wong AS Geng L Hayday A Danska JS . gammadelta T cells are essential effectors of type 1 diabetes in the nonobese diabetic mouse model. J Immunol. (2013) 190:5392–401. doi: 10.4049/jimmunol.1203502 23626013 PMC3836168

[63] Liu R Zhang J Chen S Xiao Y Hu J Zhou Z . Intestinal mucosal immunity and type 1 diabetes: Non-negligible communication between gut and pancreas. Diabetes Obes Metab. (2025) 27:1045–64. doi: 10.1111/dom.16101 PMC11802406 39618164

[64] Greenbaum CJ Nepom GT Wood-Heickman LK Wherrett DK DiMeglio LA Herold KC . Evolving concepts in pathophysiology, screening, and prevention of type 1 diabetes: report of diabetes mellitus interagency coordinating committee workshop. Diabetes. (2024) 73:1780–90. doi: 10.2337/dbi24-0020 39167668 PMC11493760

[65] Roep BO Peakman M . Diabetogenic T lymphocytes in human Type 1 diabetes. Curr Opin Immunol. (2011) 23:746–53. doi: 10.1016/j.coi.2011.10.001 22051340

[66] Insel RA Dunne JL Atkinson MA Chiang JL Dabelea D Gottlieb PA . Staging presymptomatic type 1 diabetes: a scientific statement of JDRF, the Endocrine Society, and the American Diabetes Association. Diabetes Care. (2015) 38:1964–74. doi: 10.2337/dc15-1419 26404926 PMC5321245

[67] Ziegler AG Rewers M Simell O Simell T Lempainen J Steck A . Seroconversion to multiple islet autoantibodies and risk of progression to diabetes in children. Jama. (2013) 309:2473–9. doi: 10.1001/jama.2013.6285 23780460 PMC4878912

[68] van Lummel M van Veelen PA Zaldumbide A de Ru A Janssen GM Moustakas AK . Type 1 diabetes-associated HLA-DQ8 transdimer accommodates a unique peptide repertoire. J Biol Chem. (2012) 287:9514–24. doi: 10.1074/jbc.m111.313940 22184118 PMC3308765

[69] Gonzalez-Duque S Azoury ME Colli ML Afonso G Turatsinze JV Nigi L . Conventional and Neo-antigenic Peptides Presented by beta Cells Are Targeted by Circulating Naive CD8+ T Cells in Type 1 Diabetic and Healthy Donors. Cell Metab. (2018) 28:946–60 e6. doi: 10.1016/j.cmet.2018.07.007 30078552

[70] Ziegler AG Cengiz E Kay TWH . The future of type 1 diabetes therapy. Lancet. (2025) 406:1520–34. doi: 10.1016/s0140-6736(25)01438-2 40983070

[71] Wong FS Pearson JA Wen L . Is the NOD mouse a good model for type 1 diabetes? Diabetologia. (2026) 69:3–19. doi: 10.1007/s00125-025-06579-0 PMC12686005 41206392

[72] Rigby MR DiMeglio LA Rendell MS Felner EI Dostou JM Gitelman SE . Targeting of memory T cells with alefacept in new-onset type 1 diabetes (T1DAL study): 12 month results of a randomised, double-blind, placebo-controlled phase 2 trial. Lancet Diabetes Endocrinol. (2013) 1:284–94. doi: 10.1016/s2213-8587(13)70111-6 24622414 PMC3957186

[73] Jerram ST Yang JHM Williams E Domingo-Vila C Liu YF Peakman M . Subcutaneous abatacept in new onset type 1 diabetes: clinical and immunological effects. Diabetes Metab Res Rev. (2025) 41:e70074. doi: 10.1002/dmrr.70074 40790833 PMC12340168

[74] Beese SE Price MJ Tomlinson C Sharma P Harris IM Adriano A . A systematic review and network meta-analysis of interventions to preserve insulin-secreting beta cell function in people newly diagnosed with type 1 diabetes: results from randomised controlled trials of immunomodulatory therapies. BMC Med. (2025) 23:351. doi: 10.1186/s12916-025-04201-z 40598585 PMC12211534

[75] Bougneres PF Carel JC Castano L Boitard C Gardin JP Landais P . Factors associated with early remission of type I diabetes in children treated with cyclosporine. N Engl J Med. (1988) 318:663–70. doi: 10.1056/nejm198803173181103 3125434

[76] Feutren G Papoz L Assan R Vialettes B Karsenty G Vexiau P . Cyclosporin increases the rate and length of remissions in insulin-dependent diabetes of recent onset. Results of a multicentre double-blind trial. Lancet. (1986) 2:119–24. doi: 10.1016/s0140-6736(86)91943-4 2873396

[77] Stiller CR Dupré J Gent M Jenner MR Keown PA Laupacis A . Effects of cyclosporine immunosuppression in insulin-dependent diabetes mellitus of recent onset. Science. (1984) 223:1362–7. doi: 10.1126/science.6367043 6367043

[78] Bach J-F . Insulin-dependent diabetes mellitus as an autoimmune disease. Endocr Rev. (1994) 15:516–42. doi: 10.1210/edrv-15-4-516 7988484

[79] Smith JA Tso JY Clark MR Cole MS Bluestone JA . Nonmitogenic anti-CD3 monoclonal antibodies deliver a partial T cell receptor signal and induce clonal anergy. J Exp Med. (1997) 185:1413–22. doi: 10.1084/jem.185.8.1413 9126922 PMC2196281

[80] Ngoenkam J Schamel WW Pongcharoen S . Selected signalling proteins recruited to the T-cell receptor-CD3 complex. Immunology. (2018) 153:42–50. doi: 10.1111/imm.12809 PMC5721247 28771705

[81] Andris F Van Mechelen M De Mattia F Baus E Urbain J Leo O . Induction of T cell unresponsiveness by anti-CD3 antibodies occurs independently of co-stimulatory functions. Eur J Immunol. (1996) 26:1187–95. doi: 10.1002/eji.1830260534 8647186

[82] Jenkins MK Chen CA Jung G Mueller DL Schwartz RH . Inhibition of antigen-specific proliferation of type 1 murine T cell clones after stimulation with immobilized anti-CD3 monoclonal antibody. J Immunol. (1990) 144:16–22. doi: 10.4049/jimmunol.144.1.16 2153162

[83] Boardman DA Philippeos C Fruhwirth GO Ibrahim MA Hannen RF Cooper D . Expression of a chimeric antigen receptor specific for donor HLA class I enhances the potency of human regulatory T cells in preventing human skin transplant rejection. Am J Transplant. (2017) 17:931–43. doi: 10.1111/ajt.14185 28027623

[84] Herold KC Gitelman SE Gottlieb PA Knecht LA Raymond R Ramos EL . Teplizumab: A Disease-Modifying Therapy for Type 1 Diabetes That Preserves beta-Cell Function. Diabetes Care. (2023) 46:1848–56. doi: 10.2337/dc23-0675 37607392 PMC10545553

[85] Long SA Thorpe J Herold KC Ehlers M Sanda S Lim N . Remodeling T cell compartments during anti-CD3 immunotherapy of type 1 diabetes. Cell Immunol. (2017) 319:3–9. doi: 10.1016/j.cellimm.2017.07.007 28844471 PMC5614459

[86] Ramos EL Dayan CM Chatenoud L Sumnik Z Simmons KM Szypowska A . Teplizumab and beta-Cell Function in Newly Diagnosed Type 1 Diabetes. N Engl J Med. (2023) 389:2151–61. doi: 10.1056/nejmoa2308743 37861217

[87] U.S. Food and Drug Administration . FDA approves new indication for tzield (teplizumab) for certain pediatric patients with recently diagnosed stage 3 type 1 diabetes. (2026).

[88] Pescovitz MD Greenbaum CJ Krause-Steinrauf H Becker DJ Gitelman SE Goland R . Rituximab, B-lymphocyte depletion, and preservation of beta-cell function. N Engl J Med. (2009) 361:2143–52. doi: 10.1056/nejmoa0904452 19940299 PMC6410357

[89] Adams AB Ford ML Larsen CP . Costimulation blockade in autoimmunity and transplantation: the CD28 pathway. J Immunol. (2016) 197:2045–50. doi: 10.4049/jimmunol.1601135 27591335 PMC5370073

[90] Rudd CE Taylor A Schneider H . CD28 and CTLA-4 coreceptor expression and signal transduction. Immunol Rev. (2009) 229:12–26. doi: 10.1111/j.1600-065x.2009.00770.x 19426212 PMC4186963

[91] Glatigny S Hollbacher B Motley SJ Tan C Hundhausen C Buckner JH . Abatacept targets T follicular helper and regulatory T cells, disrupting molecular pathways that regulate their proliferation and maintenance. J Immunol. (2019) 202:1373–82. doi: 10.4049/jimmunol.1801425 30683697 PMC6481683

[92] Orban T Bundy B Becker DJ DiMeglio LA Gitelman SE Goland R . Co-stimulation modulation with abatacept in patients with recent-onset type 1 diabetes: a randomised, double-blind, placebo-controlled trial. Lancet. (2011) 378:412–9. doi: 10.1016/s0140-6736(11)60886-6 21719096 PMC3462593

[93] Nikolic T Suwandi JS Wesselius J Laban S Joosten AM Sonneveld P . Tolerogenic dendritic cells pulsed with islet antigen induce long-term reduction in T-cell autoreactivity in type 1 diabetes patients. Front Immunol. (2022) 13:1054968. doi: 10.3389/fimmu.2022.1054968 36505460 PMC9728525

[94] Postigo-Fernandez J Firdessa-Fite R Creusot RJ . Preclinical evaluation of a precision medicine approach to DNA vaccination in type 1 diabetes. Proc Natl Acad Sci USA. (2022) 119:e2110987119. doi: 10.1073/pnas.2110987119 35385352 PMC9169641

[95] Postigo-Fernandez J Creusot RJ . A multi-epitope DNA vaccine enables a broad engagement of diabetogenic T cells for tolerance in Type 1 diabetes. J Autoimmun. (2019) 98:13–23. doi: 10.1016/j.jaut.2018.11.003 30454875 PMC6389442

[96] Liu YF Powrie J Arif S Yang JHM Williams E Khatri L . Immune and Metabolic Effects of Antigen-Specific Immunotherapy Using Multiple beta-Cell Peptides in Type 1 Diabetes. Diabetes. (2022) 71:722–32. doi: 10.2337/db21-0728 35073398 PMC8965665

[97] Hannelius U Beam CA Ludvigsson J . Efficacy of GAD-alum immunotherapy associated with HLA-DR3-DQ2 in recently diagnosed type 1 diabetes. Diabetologia. (2020) 63:2177–81. doi: 10.1007/s00125-020-05227-z 32754804 PMC7476912

[98] Beam CA MacCallum C Herold KC Wherrett DK Palmer J Ludvigsson J . GAD vaccine reduces insulin loss in recently diagnosed type 1 diabetes: findings from a Bayesian meta-analysis. Diabetologia. (2017) 60:43–9. doi: 10.1007/s00125-016-4122-1 PMC5856235 27704166

[99] Chen J Hu Y Chen Y Zhou Z Shen Y Wang Y . LNP-mRNA vaccine prevents type 1 diabetes in non-obese diabetes mice. J Control Release. (2024) 375:513–23. doi: 10.1016/j.jconrel.2024.09.020 39278354

[100] Denroche HC Pallo LP Chen S Ng V Zaifman J Ferrad M . 2003-LB: An mRNA immunotherapy for type 1 diabetes. Diabetes. (2025) 74:2003–LB. doi: 10.2337/db25-2003-lb

[101] Attias M Al-Aubodah T Piccirillo CA . Mechanisms of human FoxP3(+) T(reg) cell development and function in health and disease. Clin Exp Immunol. (2019) 197:36–51. doi: 10.1111/cei.13290 30864147 PMC6591147

[102] Tenspolde M Zimmermann K Weber LC Hapke M Lieber M Dywicki J . Regulatory T cells engineered with a novel insulin-specific chimeric antigen receptor as a candidate immunotherapy for type 1 diabetes. J Autoimmun. (2019) 103:102289. doi: 10.1016/j.jaut.2019.05.017 31176558

[103] Kakabadse D Chen D Fishman S Weinstein-Marom H Davies J Wen L . Regulatory CD4(+) T cells redirected against pathogenic CD8(+) T cells protect NOD mice from development of autoimmune diabetes. Front Immunol. (2024) 15:1463971. doi: 10.3389/fimmu.2024.1463971 39351219 PMC11439686

[104] Stamatouli AM Quandt Z Perdigoto AL Clark PL Kluger H Weiss SA . Collateral damage: Insulin-dependent diabetes induced with checkpoint inhibitors. Diabetes. (2018) 67:1471–80. doi: 10.2337/dbi18-0002 29937434 PMC6054443

[105] Chen J Hou X Yang Y Wang C Zhou J Miao J . Immune checkpoint inhibitors-induced diabetes mellitus (review). Endocrine. (2024) 86:451–8. doi: 10.1007/s12020-024-03942-w 38955861

[106] Fife BT Bluestone JA . Control of peripheral T-cell tolerance and autoimmunity via the CTLA-4 and PD-1 pathways. Immunol Rev. (2008) 224:166–82. doi: 10.1111/j.1600-065x.2008.00662.x 18759926

[107] Walker LS Sansom DM . The emerging role of CTLA4 as a cell-extrinsic regulator of T cell responses. Nat Rev Immunol. (2011) 11:852–63. doi: 10.1038/nri3108 22116087

[108] Nikolic T Zwaginga JJ Uitbeijerse BS Woittiez NJ de Koning EJ Aanstoot HJ . Safety and feasibility of intradermal injection with tolerogenic dendritic cells pulsed with proinsulin peptide-for type 1 diabetes. Lancet Diabetes Endocrinol. (2020) 8:470–2. doi: 10.1016/s2213-8587(20)30104-2 32723484

[109] Stoeckius M Hafemeister C Stephenson W Houck-Loomis B Chattopadhyay PK Swerdlow H . Simultaneous epitope and transcriptome measurement in single cells. Nat Methods. (2017) 14:865–8. doi: 10.1038/nmeth.4380 28759029 PMC5669064

[110] Hauser SL Bar-Or A Comi G Giovannoni G Hartung HP Hemmer B . Ocrelizumab versus interferon beta-1a in relapsing multiple sclerosis. N Engl J Med. (2017) 376:221–34. doi: 10.1056/nejmoa1601277 28002679

[111] Postow MA Sidlow R Hellmann MD . Immune-related adverse events associated with immune checkpoint blockade. N Engl J Med. (2018) 378:158–68. doi: 10.1056/nejmra1703481 29320654

